# *Pueraria mirifica*: a critical review of the ethnobotany, phytochemistry, pharmacology and challenges in standardization and safety

**DOI:** 10.3389/fphar.2026.1820644

**Published:** 2026-06-05

**Authors:** Haiqin Zeng, Juan Wen, Minqi Chen, Zhendong Qiu, Shiping Huang, Erwei Hao, Zhengcai Du, Chun Yao, Xiaotao Hou, Jiagang Deng

**Affiliations:** 1 Guangxi Key Laboratory of Efficacy Study on Chinese Materia Medica, Guangxi University of Chinese Medicine, Nanning, China; 2 Faculty of Pharmacy, Guangxi University of Chinese Medicine, Nanning, China; 3 Guangxi Key Laboratory of TCM Formulas Theory and Transformation for Damp Diseases, Guangxi University of Chinese Medicine, Nanning, China; 4 University Engineering Research Center of Reutilization of Traditional Chinese Medicine Resources, Guangxi University of Chinese Medicine, Nanning, China

**Keywords:** chemical metabolites, clinical applications, ethnopharmacology, menopausal syndrome, pharmacological mechanisms, phytoestrogens, *Pueraria mirifica*, quality control

## Abstract

**Background:**

*Pueraria candollei* var. *mirifica* (Airy Shaw and Suvat.) Niyomdham (*Pueraria mirifica*), locally known as “White Kwao Krua” or “white kudzu”, is a renowned medicinal botanical drug indigenous to Southeast Asia, particularly Thailand and Myanmar. It has long been utilized in ethnomedicine for estrogen-related conditions and age-associated functional decline. This review critically evaluates the nexus between its botanical characteristics, traditional ethnopharmacological uses, and contemporary evidence regarding its therapeutic potential.

**Materials and Methods:**

A structured literature search was conducted across multiple international and Chinese databases up to 2025, with screening based on predefined inclusion and exclusion criteria focusing on botanical identity, chemical composition, pharmacological activity, toxicological evidence, and clinical findings. Taxonomic validity and geographic distribution were verified using Plants of the World Online (POWO) and the Global Biodiversity Information Facility (GBIF). The names and chemical formulas of secondary metabolites were verified using the PubChem database, and the structures were drawn using ChemDraw 22.2.

**Results:**

Phytochemical studies on the rhizomes of *P. mirifica* showed that it contains 106 kinds of secondary metabolites, including benzopyrans, isoflavones and their glycosides, coumarins, steroids, fatty acids and their derivatives, and other metabolites. Pharmacological evidence demonstrates that *P. mirifica* exhibits potent estrogenic, anti-osteoporotic, and antioxidant activities, alongside neuroprotective and immunomodulatory effects. Clinical data suggest that its high concentration of potent phytoestrogens effectively mitigates vasomotor symptoms and urogenital syndromes in menopausal women, while positively influencing lipid metabolism and bone mineral density.

**Conclusion:**

*P. mirifica* represents a high-potency phytoestrogenic candidate for menopause management. Nevertheless, the translation from traditional use to clinical standardization is hindered by a lack of rigorous pharmacokinetic data and inconsistent quality control of metabolites. Future research should move beyond descriptive summaries toward mechanistic studies that define long-term safety margins and standardized dose–response relationships in order to ensure therapeutic reliability.

## 1 Introduction

Since the 20th century, global attention toward perimenopausal symptoms and their long-term complications has intensified. Hormone Replacement Therapy (HRT) remains the gold standard for clinical intervention (Kandale et al., 2025); however, its long-term application is frequently constrained by concerns regarding elevated risks of breast cancer and cardiovascular events (Vigneswaran and Hamoda, 2022; Intharuksa et al., 2025). Consequently, there is an urgent global demand for safe, plant-derived alternatives. *Pueraria candollei* var. *mirifica* (Airy Shaw and Suvat.) Niyomdham (*Pueraria mirifica*), commonly known as “White Kwao Krua”, has emerged as a premier candidate due to its unique profile of potent phytoestrogens, notably the chromene derivatives miroestrol and deoxymiroestrol (Chansakaow et al., 2000).

*Pueraria candollei* var. *mirifica* (Airy Shaw and Suvat.) Niyomdham (*P. mirifica*), commonly known as White Kwao Krua or white kudzu, is a perennial climbing plant. Historically, *P. mirifica* has been used for more than a century, primarily as a rejuvenating tonic to alleviate age-related ailments in menopausal women (Maruyama et al., 2014). It is essential to distinguish *P. mirifica* from its congener, *Pueraria montana* var. *lobata*. While the latter is widely used in East Asia for cardiovascular disorders and febrile diseases, it lacks the specific benzopyranoid scaffold that grants *P. mirifica* its exceptional estrogenic potency (Malaivijitnond, 2012). Despite its burgeoning commercial presence in the global nutraceutical and cosmetic markets, the scientific evidence supporting *P. mirifica* remains fragmented. Current literature often provides descriptive summaries that lack a rigorous, critical assessment of study quality, molecular mechanisms, and the translational strength of clinical data.

**FIGURE 1:**
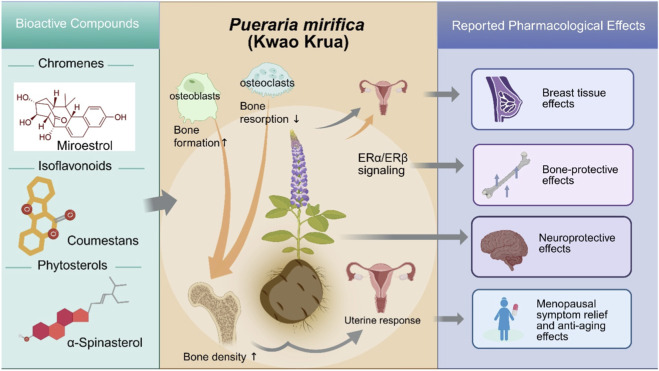
Integrative pharmacological framework of *Pueraria mirifica*. This schematic illustrates the major bioactive constituents of *Pueraria mirifica* and their reported pharmacological effects. The central panel highlights estrogen receptor (ERα/ERβ) signaling as a key proposed mechanistic pathway. In the bone-related context, *Pueraria mirifica* is shown to promote osteoblast-mediated bone formation and inhibit osteoclast-mediated bone resorption (indicated by directional arrows), thereby contributing to increased bone density. The right panel summarizes reported pharmacological effects, including breast tissue effects, bone-protective effects, neuroprotective effects, and menopausal symptom relief and anti-aging effects (Created with BioRender.com).

This review aims to provide a critical and systematic synthesis of *P. mirifica*, bridging its ethnopharmacological heritage with contemporary scientific evidence. Rather than offering a purely descriptive compilation, this review evaluates the strength, limitations, and translational relevance of the available evidence across ethnobotanical, phytochemical, pharmacological, toxicological, and clinical domains. Particular emphasis is placed on distinguishing comprehensive chemical reporting from pharmacologically prioritized constituents, separating mechanistic plausibility from experimentally supported effects, and differentiating preclinical findings from clinically actionable evidence. In addition, this review identifies key sources of inconsistency, including phytochemical variability, formulation heterogeneity, limited pharmacokinetic characterization, and insufficient long-term clinical validation, all of which currently constrain the development of *P. mirifica* as a standardized evidence-based botanical drug. To provide an integrated overview, the major bioactive metabolites of *P. mirifica*, their proposed estrogen receptor-related mechanisms, and the reported pharmacological effects are summarized in Figure 1.

## 2 Review methodology

**FIGURE 2:**
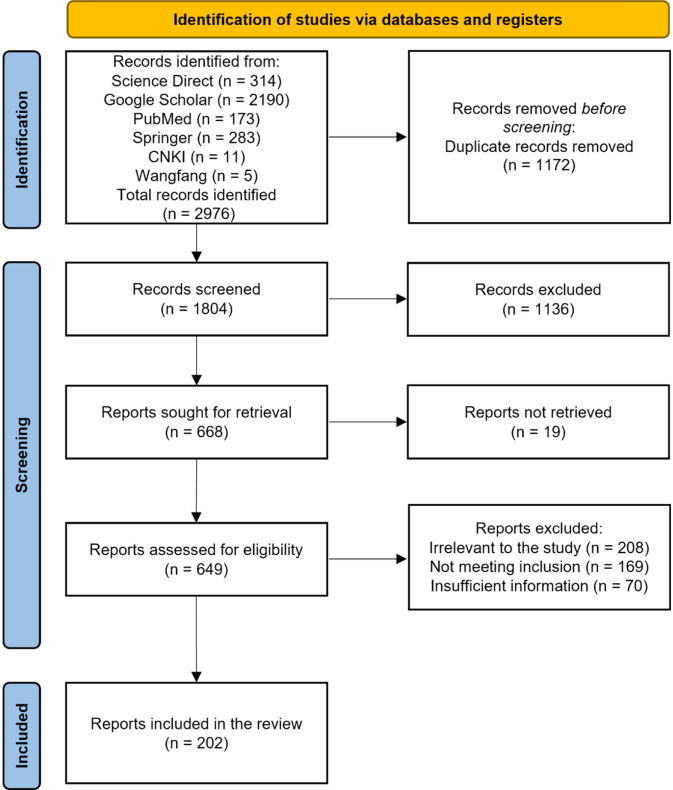
Literature screening and study selection process. This flow diagram summarizes the literature identification, duplicate removal, screening, eligibility assessment, and final inclusion workflow used in this review. Numbers shown in each box indicate the records or reports retained or excluded at each stage of the selection process.

To ensure transparency and reproducibility, a structured approach was employed for literature identification, screening, and evaluation. A database search was conducted across ScienceDirect, Google Scholar, PubMed, Springer, CNKI, and Wanfang using combinations of the following search terms: (“*Pueraria mirifica*” OR “White Kwao Krua” OR “Kwao Krua”) AND (phytochemistry OR chemical metabolite OR metabolite OR pharmacology OR biological activity OR clinical trial OR menopause). The search initially identified 2,976 records potentially relevant to the topic. After duplicate removal and screening according to the predefined inclusion and exclusion criteria, 202 reports were finally included in this review. The retrieved literature was further categorized into ethnobotanical, phytochemical, pharmacological, toxicological, and clinical domains. Evidence interpretation was informed not only by topic relevance but also by study type and methodological detail, with explicit consideration of whether findings were derived from *in vitro* experiments, animal studies, or human investigations. Particular attention was given to study design clarity, sample size, dosage information, control settings, formulation characterization, and the extent to which reported findings supported mechanistic interpretation or clinical translation. The taxonomic validity of *P. mirifica* was verified using authoritative taxonomic sources, and its botanical distribution was cross-checked against reliable biodiversity databases. In addition, the chemical nomenclature and molecular structures of the identified compounds were verified using the PubChem database and ChemDraw (version 22.2). Accordingly, this article should be understood as a structured and critical evidence synthesis rather than a formal systematic review with meta-analysis. The overall literature retrieval, screening, and study selection workflow is summarized in Figure 2.

## 3 Botanical profile and traditional use

### 3.1 Taxonomy and vernacular names

Accurate taxonomic identification is essential for distinguishing *P. mirifica* from closely related taxa and for ensuring consistency across ethnobotanical, phytochemical, and pharmacological studies. According to Plants of the World Online (POWO), *P. mirifica* belongs to the family Fabaceae and the genus *Pueraria*. In addition to its formal taxonomic placement, the species is known by different vernacular names across Southeast Asia, reflecting regional linguistic variation and its long-standing traditional use. Reported local names of *P. mirifica* across Southeast Asia are summarized in Table 1.

**TABLE 1:** Reported local names of *P. mirifica* across Southeast Asia. Local names are presented as reported in the cited sources and may vary across regions and dialects. Country/region entries indicate the geographic contexts in which these vernacular names have been reported.

Country/Region	Local name (s)	References
Thailand	Kwao Krua; Kwao Keur	(Kashemsanta et al., 1952)
Thailand-North	Jan Krua	(Su, 2017)
Thailand-Northeast	Tan Krua	(Su, 2017)
Thailand-South	Tan Chom Thong	(Su, 2017)
Myanmar	Paukse; UU mhwun ping; U mhwun (pu ti)	Multilingual Multiscript Plant Name Database
Laos	No indexed local name identified	-
Vietnam	Sâm tố nữ	-

### 3.2 Geographic distribution

**FIGURE 3:**
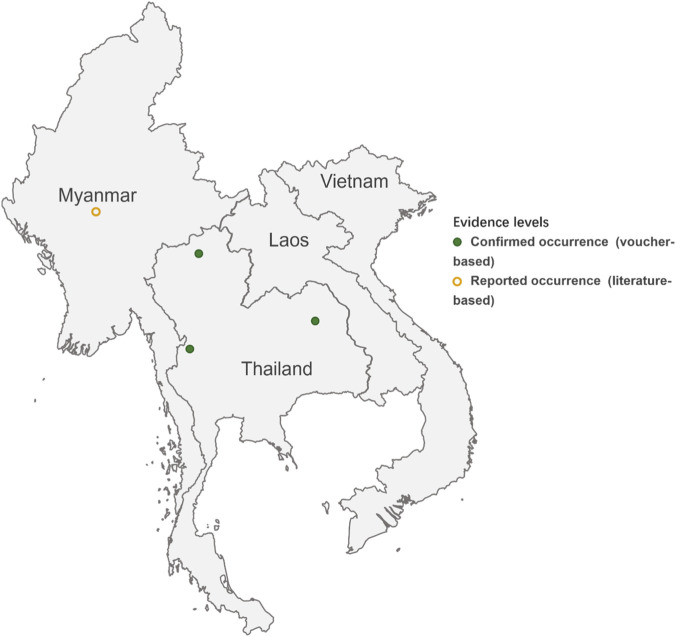
Geographic distribution and evidence levels of *Pueraria mirifica* in mainland Southeast Asia. The map summarizes the currently available evidence for the geographic distribution of *Pueraria mirifica* in mainland Southeast Asia. Green circles indicate confirmed occurrences supported by voucher-based or other primary evidence, whereas orange circles indicate literature-based reported occurrences without specimen confirmation. The mapped points are schematic and represent country-level or broad regional evidence rather than exact localities. Thailand represents the principal area of confirmed occurrence, whereas records from Myanmar, Laos, and Vietnam are retained as reported occurrences with lower evidential confidence. Country labels indicate the geographic scope of the currently available evidence. The map is intended to provide a conceptual overview rather than exhaustive occurrence mapping.

The currently available evidence indicates that *P. mirifica* is centered in mainland Southeast Asia, with Thailand representing the principal area of confirmed native occurrence. The species was originally described from Thailand, and subsequent studies have reported its broad distribution in Thai deciduous forests, particularly in the northern, western, and northeastern regions, with additional collections from multiple provinces across the country (Kashemsanta et al., 1952; Malaivijitnond, 2012; Cherdshewasart et al., 2008). By contrast, records from neighboring countries remain less well supported. Occurrence in Myanmar has been reported in the literature, but it has not been confirmed to the same taxonomic or floristic standard as records from Thailand (Malaivijitnond, 2012). Reports referring to Laos and Vietnam are also available in secondary literature or local studies, but these should be interpreted cautiously because the supporting evidence is weaker than that for Thailand (Rani et al., 2022a; Vui et al., 2019). Accordingly, Thailand is treated here as the confirmed occurrence area, whereas Myanmar, Laos, and Vietnam are shown as reported occurrences with lower evidential confidence. The geographic distribution and evidence levels of *P. mirifica* in mainland Southeast Asia are summarized in Figure 3.

### 3.3 Gross morphology

**FIGURE 4:**
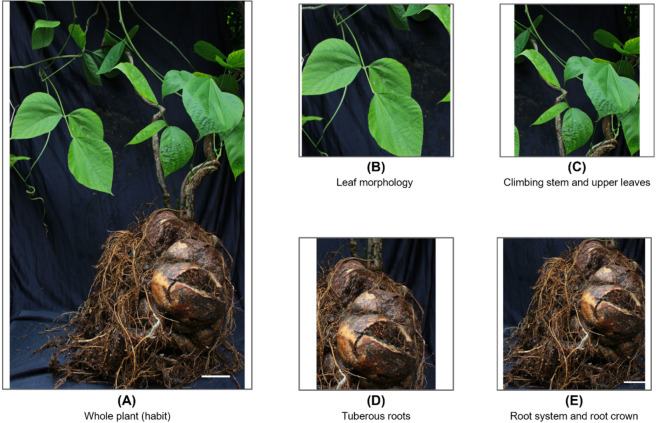
Morphological characteristics of *Pueraria mirifica*. **(A)** Whole-plant habit; **(B)** leaf morphology; **(C)** climbing stem and upper leaves; **(D)** tuberous roots; **(E)** root system and root crown. Adapted from Suntichaikamolkul et al. (2019), Additional file 1: Figure S1, under the Creative Commons Attribution 4.0 International License (CC BY 4.0).

Morphological characterization provides an important basis for species recognition and helps support the botanical authenticity of materials used in phytochemical and pharmacological investigations. *Pueraria mirifica* is a woody climber that can reach up to 12 m in length and is characterized by a twining habit, slender branched stems, trifoliate leaves, and prominent tuberous roots. The stems are glabrous, ribbed, and become flaking with age. The leaves are pinnately trifoliate, with acuminate apices, broadly ovate to obtuse bases, entire margins, and a pubescent surface. The underground organs are globose to ovoid tuberous roots, typically 5–40 cm in diameter, with a pale yellow, glabrous outer surface and white internal tissue that becomes progressively lignified with age. The gross morphological characteristics of *P. mirifica*, including its climbing habit, trifoliate leaves, and tuberous roots, are shown in Figure 4.

### 3.4 Ethnopharmacological relevance and traditional knowledge

#### 3.4.1 Botanical origin and taxonomic evolution of *Pueraria mirifica*

Historical records from Myanmar indicate that *P. mirifica* was traditionally distinguished from related *P. mirifica* materials on the basis of vegetative morphology and root coloration. These records specifically emphasized the medicinal importance of *P. mirifica* as the therapeutically valued form. This text specifically detailed the morphological distinctions between *P. mirifica* and *Butea superba*, highlighting the medicinal value of the former as a therapeutic agent or health supplement.

The taxonomic history of *P. mirifica* has undergone several revisions, reflecting earlier uncertainty in species identification and later clarification based on morphological and botanical evidence. After initial confusion with related taxa, the species was ultimately recognized as *Pueraria mirifica* and later reclassified as *Pueraria candollei* var. *mirifica* to distinguish it from closely related forms within the genus. This taxonomic clarification is not merely historical; it has direct relevance to phytochemical authentication, quality control, and the interpretation of pharmacological studies, because inconsistent nomenclature in the literature may obscure the true botanical identity of the materials investigated.

#### 3.4.2 Traditional applications and ethnopharmacological context

In ethnobotanical practice, harvested *P. mirifica* undergo a rigorous preparation process, including removal of impurities, thorough washing, peeling of the periderm, and a secondary rinse. The cleaned roots are then sliced thinly, dried (either *via* solar or oven drying), and pulverized into a fine powder. Alternatively, the powder is mixed with honey to form boluses (pills) for precise dosing and improved formulation stability.

According to the *P. mirifica* Prescription, *P. mirifica* can be administered as a monotherapy or in polyherbal formulations with milk, honey, or other traditional Thai botanical drugs. Its traditional therapeutic indications include: rejuvenation, improved sleep, enhanced physical strength, promoting appetite, breast enlargement, preventing hair loss or graying, and memory enhancement.

Historical texts specify a dosage range of 30–500 mg/day, recommending a gradual escalation from low to high doses. These traditional applications provide an important ethnopharmacological rationale for subsequent scientific investigation, particularly in relation to women’s health, menopausal symptom management, and age-associated functional decline (Maruyama et al., 2014). However, the translational pathway from traditional use to modern application remains incomplete. Specifically, traditional indications do not directly identify the active constituents responsible for these effects, nor do they resolve questions regarding dose standardization, tissue selectivity, long-term safety, or formulation consistency. For this reason, the following sections examine how phytochemical evidence, mechanistic pharmacology, and clinical findings collectively support—yet also limit—the modern therapeutic interpretation of *P. mirifica*.

#### 3.4.3 Comparative botanical analysis and identification

Based on traditional classifications, *P. mirifica* is frequently discussed alongside two other regionally significant botanicals: *B. superba* and *Mucuna collettii*. While all three species are utilized in Southeast Asia for kidney tonification and share superficial morphological similarities, their botanical origins, chemical profiles, and therapeutic applications differ significantly. Notably, *P. mirifica* is primarily indicated for female health due to its unique phytoestrogen content; in contrast, the latter two species are traditionally administered for male vitality. Phytochemical investigations have further delineated these distinctions, revealing that *B. superba* lacks the potent chromene miroestrol, which is diagnostic for *P. mirifica*, though it possesses higher concentrations of the isoflavones daidzein and genistein (Manosroi and Manosroi, 2005). Such chemical divergence highlights the risks of substitution, especially as identification becomes increasingly challenging when these materials are marketed in powdered or processed forms. To address the limitations of traditional organoleptic identification and ensure consumer safety, molecular diagnostic techniques—including polymerase chain reaction–restriction fragment length polymorphism (PCR-RFLP) (Wiriyakarun et al., 2013) and Amplification Refractory Mutation System (ARMS) (Intharuksa et al., 2020)—have been rigorously implemented to resolve inter-species ambiguities.

Within the Pueraria genus, *P. candollei* and its variety var. *mirifica* are highly susceptible to adulteration due to their nearly identical vegetative morphology. Early literature suggested that differentiation was only possible during the flowering period (March-April) by observing inflorescence density and pod pubescence (Yusakul et al., 2011). However, subsequent research has revealed fundamental differences at both the chemical and genetic levels: (1) Phytohormonal Profile: var. *mirifica* contains the unique marker mirificin and significantly higher concentrations of puerarin, with metabolic pathways that are highly sensitive to environmental induction (Korsangruang et al., 2010; Boonsnongcheep et al., 2010). (2) Molecular Markers: While standard PCR-RFLP may lack the resolution for this specific variety (Wiriyakarun et al., 2013), recent studies identify a single nucleotide polymorphism (G/C variation) at position 702 of the matK gene and stipule length (>0.9 cm vs. <0.4 mm) as definitive diagnostic markers (Charoensup et al., 2024). Integrating morphological, biochemical, and molecular approaches is essential to ensure clinical efficacy and safety. Furthermore, a literature review reveals that some studies still erroneously use the umbrella term *P. candollei* when the subject is actually *P. candollei* var. *mirifica*. We emphasize the urgent need for standardized identification and unified nomenclature to ensure the scientific rigor of future research.

### 3.5 Quantitative variation in chemical constituents: influence of genetics, geography, and seasonality

Traditional knowledge underscores the critical importance of harvest timing and processing methods to ensure the therapeutic efficacy of *P. mirifica*. Recent quantitative studies have validated these claims, revealing that the chemical profile of *P. mirifica* is a dynamic result of complex interactions between genetic background (cultivars), geographic origin, seasonal fluctuations, and extraction technologies. This variability directly influences the amounts of key phytoestrogens (e.g., miroestrol, deoxymiroestrol), which are often used as markers for product potency and activity, making standardized quality control essential in commercial production. Without rigorous authentication and quantification of these bioactive constituents, products derived from different sources can vary widely in their chemical profile, leading to inconsistent pharmacological effects (Rani et al., 2022). Quality assessment frameworks for herbal products emphasize the need to evaluate authentication, compound quantification, safety, and efficacy before formulation.

#### 3.5.1 Geographic and genetic chemovariation

Quantitative analysis demonstrates substantial inter-provincial and intra-provincial variation in phytoestrogen content across Thailand. Total isoflavone levels can vary by over 10-fold between regions, with the highest concentrations reported in Kanchanaburi province (198.29 mg/100 g) and significantly lower levels in Nan province (18.61 mg/100 g) (Cherdshewasart et al., 2007). Even within a single province such as Chiang Mai, a 3-fold variation exists between districts (e.g., 240.09 mg/100 g in Doi Tao vs. 76.52 mg/100 g in Chaiprakarn), confirming a clinal pattern of chemical distribution (Suwanvijitr et al., 2010).

Beyond geographic factors, distinct cultivars (e.g., *P. mirifica* -I to *P. mirifica* -V) exhibit divergent “chemovarieties” even when cultivated under identical environmental conditions; for instance, *P. mirifica* -I consistently demonstrates superior estrogenic activity and isoflavonoid accumulation (up to 1007.47 ± 72.22 mg/100 g) compared to *P. mirifica* -II (Cherdshewasart and Sriwatcharakul, 2007).

Recent multi-omic integrative studies further elucidate these phenotypic discrepancies, revealing that concatenated chloroplast DNA barcodes (including matK, rpoC1, trnH-psbA, and rps16) provide superior resolution for discriminating *P. mirifica* cultivars compared to nuclear internal transcribed spacer (ITS) markers. This genetic divergence is closely coupled with differential gene expression; specifically, high-performing cultivars (such as “NA”) exhibit a coordinated upregulation of key biosynthetic genes—including IFS, CYP81E63, and the novel UGT2—within the tubers, directly correlating with elevated daidzein and puerarin levels. Such findings underscore that precise cultivar selection, guided by integrated barcoding and transcriptomic profiling, is indispensable for ensuring the quality, safety, and standardized therapeutic efficacy of *P. mirifica* products (Huynh et al., 2024).

#### 3.5.2 Seasonal dynamics and metabolic flux

The accumulation of bioactive metabolites is highly sensitive to seasonal changes, driven by the differential expression of synthetic enzymes such as UDP-glycosyltransferase (UGT) and CHI (Jungsukcharoen et al., 2016). (1) Summer (April): Generally, represents the optimal harvest window. Total isoflavones can be 15 to 37 times higher than in the rainy season. Puerarin, for instance, peaks at 228.41 ± 87.80 mg/kg in summer compared to only 14.48 ± 6.03 mg/kg in the rainy season (Cherdshewasart and Sriwatcharakul, 2007). When *P. mirifica* grew, the growth rate increased with the increase of temperature from 16 °C to 27 °C (Major et al., 1975). (2) Rainy season (October): Characterized by a shift toward aglycones, with daidzein reaching its relative peak, though total isoflavone activity is lowest due to high water content and metabolic dilution (Cherdshewasart et al., 2008). (3) Winter (February): Genistein content typically peaks during this period, and biological activity remains relatively high, comparable to summer (Cherdshewasart et al., 2008).

#### 3.5.3 Impact of extraction and processing methods

The recovery of these constituents is further dictated by the extraction methodology. Traditional ethanol extraction (80%) yields a crude recovery of approximately 5.38%, whereas sequential partitioning into dichloromethane (DCM) concentrates highly active lipophilic metabolites like miroestrol and deoxymiroestrol, despite a lower mass yield of 0.22% (Sookvanichsilp et al., 2008). Modern intensification techniques have significantly outperformed these traditional protocols; for instance, ultra-fine pulverization (100–300 mesh) combined with ultrasonic-microwave synergistic extraction (UMSE) achieves a flavonoid extraction rate of 4.86% in just 30 min (Zhu et al., 2023). Building upon these advancements, the field is shifting towards sustainable pharmacognosy through the use of Natural Deep Eutectic Solvents (NADES) and advanced analytical integration. Recent studies demonstrate that honey-based NADES (H-NADES) not only serves as an eco-friendly solvent for the high-sensitivity extraction and icELISA-based quantification of daidzin, but also facilitates the *in situ* bioconversion of daidzin into the more bioactive aglycone daidzein *via* endogenous β-glucosidase activity (Yusakul et al., 2021). Furthermore, the integration of green extraction technologies—such as enzyme-assisted and supercritical fluid extraction—with high-resolution UPLC-MS/MS and NMR platforms has been pivotal in elucidating the complex biosynthetic pathways and broad pharmacological spectrum of puerarin, encompassing its cardiovascular, neuroprotective, and osteogenic effects (Liga and Paul, 2024).

#### 3.5.4 Conclusion on material standardization

These findings highlight that material standardization is a central translational challenge in *P. mirifica* research rather than a merely technical issue. A practical quality framework should include at least five elements: authenticated botanical identity, control of raw material origin and harvest conditions, chemical fingerprinting with prioritized marker compounds, batch-to-batch consistency testing, and bioactivity-linked quality evaluation. In this context, variables such as plant age, cultivar selection, season of harvest, and post-harvest processing are not minor background factors but major determinants of metabolite composition and therefore of pharmacological consistency. Consequently, future standardization efforts should move beyond reliance on single-marker quantification and instead adopt integrated metabolic fingerprinting strategies combined with safety evaluation and pharmacokinetic characterization to support clinically reliable product development.

Importantly, this phytochemical variability is not only a compositional issue but also a translational one. Variations in cultivar, geographic origin, seasonality, and processing conditions can directly alter the relative abundance of active metabolites, thereby affecting pharmacological consistency, batch-to-batch reproducibility, comparability across studies, and ultimately the clinical reliability of *P. mirifica*-derived products. This is one of the key reasons why promising preclinical findings cannot yet be assumed to translate into uniform therapeutic outcomes in human use.

## 4 Phytochemical composition

**FIGURE 5:**
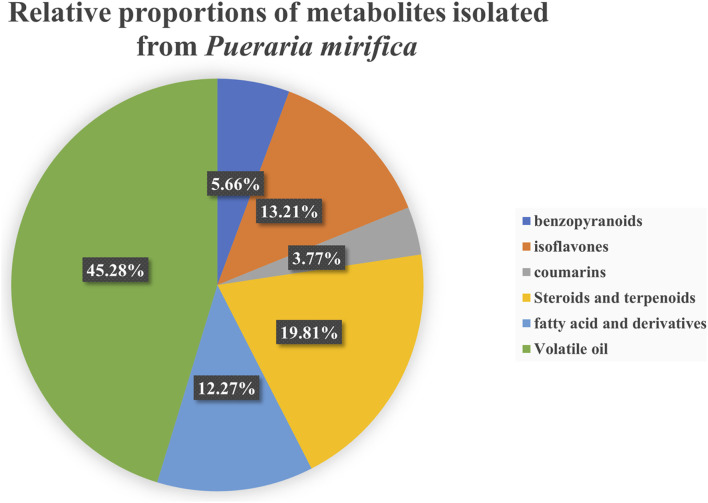
Relative proportions of major phytochemical classes identified from *Pueraria mirifica*. This pie chart summarizes the relative distribution of the reported secondary metabolites of *Pueraria mirifica* according to the principal chemical classes summarized in Table 2. These classes include benzopyranoids, isoflavones and related glycosides, coumarins, steroids and terpenoids, fatty acids and related derivatives, and volatile constituents. The percentages shown in the chart indicate the relative contribution of each phytochemical class within the currently compiled metabolite dataset.

**FIGURE 6:**
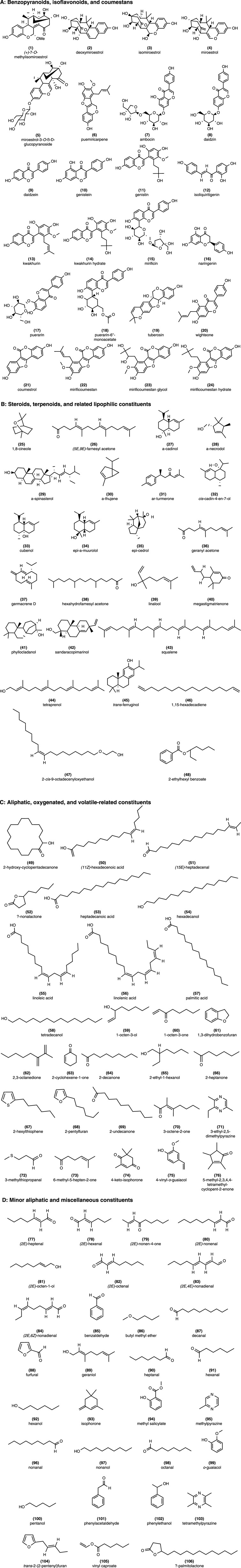
Chemical structures of reported secondary metabolites identified from *Pueraria mirifica*. Panels **(A–D)** illustrate representative chemical structures of the reported secondary metabolites isolated or identified from *Pueraria mirifica*, arranged according to structural classification. The compound numbers correspond directly to the entries summarized in Table 2, facilitating cross-reference between the structural figures and the phytochemical inventory presented in the manuscript.

*Pueraria mirifica* possesses a complex and distinctive phytochemical profile. Although it shares several common isoflavonoids with other Pueraria species, such as *Pueraria lobata*, it is distinguished by the presence of benzopyranoid constituents, particularly deoxymiroestrol and miroestrol, which are widely regarded as characteristic phytoestrogenic compounds of this species. To date, more than 100 metabolites have been identified from *P. mirifica*, reflecting substantial chemical diversity. However, not all reported constituents appear to contribute equally to its pharmacological profile. Based on current evidence, chromenes—especially deoxymiroestrol and miroestrol—together with selected isoflavonoids and related compounds such as puerarin, daidzein, genistein, and kwakhurin, are currently the most biologically relevant constituents for interpreting the reported estrogenic and downstream pharmacological effects of *P. mirifica*. Accordingly, Table 2 and Figures 5, 6 should be interpreted not only as a comprehensive phytochemical inventory, but also as a basis for pharmacological prioritization and future standardization.

**TABLE 2:** Chemical constituents identified from *P. mirifica* and their structural classification. This table summarizes the reported secondary metabolites of *P. mirifica*, including their chemical classes, molecular information, extract or fraction source, and corresponding references.

No.	Category	Compound name	Molecular formula	Extract/Fraction	PubChem CID	Reference(s)
1	benzopyranoids	(*+*)-7-*O*-methylisomiroestrol	C_21_H_24_O_6_	CH_2_Cl_2_ soluble fraction of EtOH extract	32077760	(Yusakul et al., 2021)
2	​	deoxymiroestrol	C_20_H_22_O_5_	EtOAc extract	9927999	(Yusakul et al., 2013a; Yusakul et al., 2013b)
3	​	isomiroestrol	C_20_H_22_O_6_	EtOAc extract	10089763	(Kitisripanya et al., 2016; 2017)
4	​	miroestrol	C_20_H_22_O_6_	MeOH extract	165001	(Cain, 1960)
5	​	miroestrol-3-*O*-β-D-glucopyranoside	C_26_H_32_O_11_	n-BuOH-soluble fraction of EtOH extract	N/A	(Bang et al., 2013)
6	​	puemiricarpene	C_21_H_20_O_5_	EtOAc extract	10089353	(Kashemsanta et al., 1952)
7	isoflavones	ambocin	C_26_H_28_O_14_	hydro-distillation	5491738	(Cho et al., 2014)
8	​	daidzin	C_21_H_20_O_9_	MeOH extract	107971	(Ingham et al., 1986)
9	​	daidzein	C_15_H_10_O_4_	MeOH extract	5281708	(Ingham et al., 1986)
10	​	genistein	C_15_H_10_O_5_	MeOH extract	5280961	(Ingham et al., 1986)
11	​	genistin	C_15_H_10_O_5_	MeOH extract	14504257	(Ingham et al., 1986)
12	​	isoliquiritigenin	C_15_H_12_O_4_	EtOAc-soluble fraction of EtOH extract	638278	(Cho et al., 2014)
13	​	kwakhurin	C_21_H_20_O_6_	EtOAc extract	11303135	(Tahara et al., 1987)
14	​	kwakhurin hydrate	C_21_H_22_O_7_	MeOH extract	14504257	(Ingham et al., 1989)
15	​	mirificin	C_26_H_28_O_13_	MeOH extract	21676217	(Ingham et al., 1986)
16	​	naringenin	C_15_H_12_O_5_	EtOAc-soluble fraction of EtOH extract	439246	(Cho et al., 2014)
17	​	puerarin	C_21_H_20_O_9_	MeOH extract	5281807	(Ingham et al., 1986)
18	​	puerarin-6''-monoacetate	C_23_H_22_O_10_	MeOH extract	44257208	(Ingham et al., 1986a)
19	​	tuberosin	C_20_H_18_O_5_	MeOH extract	5318770	(Chansakaow et al., 2000)
20	​	wighteone	C_20_H_18_O_5_	hydro-distillation	5281814	(Cho et al., 2014)
21	coumarins	coumestrol	C_15_H_8_O_5_	MeOH extract	5281707	(Tahara et al., 1987)
22	​	mirificoumestan	C_21_H_18_O_6_	MeOH extract	13964266	(Ingham et al., 1988)
23	​	mirificoumestan glycol	C_21_H_20_O_8_	MeOH extract	13964268	(Ingham et al., 1988)
24	​	mirificoumestan hydrate	C_21_H_20_O_7_	MeOH extract	13964267	(Ingham et al., 1988)
25	Steroids and terpenoids	1,8-cineole	C_10_H_18_O	hydro-distillation	2758	(Yagi et al., 2013)
26	​	(*5E,9E*)-farnesyl acetone	C_18_H_30_O	hydro-distillation	1711945	(Yagi et al., 2013)
27	​	α-cadinol	C_15_H_26_O	hydro-distillation	10398656	(Yagi et al., 2013)
28	​	α-necrodol	C_10_H_18_O	hydro-distillation	10909794	(Yagi et al., 2013)
29	​	α-spinasterol	C_29_H_48_O	EtOH extract	5281331	(Jeon et al., 2005)
30	​	α-thujene	C_10_H_16_	hydro-distillation	17868	(Yagi et al., 2013)
31	​	ar-turmerone	C_15_H_20_O	hydro-distillation	160512	(Yagi et al., 2013)
32	​	*cis*-cadin-4-en-7-ol	C_15_H_26_O	hydro-distillation	91746528	(Yagi et al., 2013)
33	​	cubenol	C_15_H_26_O	hydro-distillation	11770062	(Yagi et al., 2013)
34	​	epi-α-muurolol	C_15_H_26_O	hydro-distillation	3084331	(Yagi et al., 2013)
35	​	epi-cedrol	C_15_H_26_O	hydro-distillation	6713078	(Yagi et al., 2013)
36	​	geranyl acetone	C_13_H_22_O	hydro-distillation	1549778	(Yagi et al., 2013)
37	​	germacrene D	C_15_H_24_	hydro-distillation	5317570	(Yagi et al., 2013)
38	​	hexahydrofarnesyl acetone	C_18_H_36_O	hydro-distillation	10408	(Yagi et al., 2013)
39	​	linalool	C_10_H_18_O	hydro-distillation	6549	(Yagi et al., 2013)
40	​	megastigmatrienone	C_13_H_18_O	hydro-distillation	5375190	(Yagi et al., 2013)
41	​	phyllocladanol	C_20_H_34_O	hydro-distillation	44237348	(Yagi et al., 2013)
42	​	sandaracopimarinol	C_20_H_32_O	hydro-distillation	12314286	(Yagi et al., 2013)
43	​	squalene	C_30_H_50_	hydro-distillation	638072	(Yagi et al., 2013)
44	​	tetraprenol	C_20_H_34_O	hydro-distillation	65183	(Yagi et al., 2013)
45	​	*trans*-ferruginol	C_20_H_30_O	hydro-distillation	442027	(Yagi et al., 2013)
46	fatty acid and derivatives	1,15-hexadecadiene	C_16_H_30_	hydro-distillation	519908	(Yagi et al., 2013)
47	​	2-*cis*-9-octadecenyloxyethanol	C_20_H_40_O_2_	hydro-distillation	5364713	(Yagi et al., 2013)
48	​	2-ethylhexyl benzoate	C_15_H_22_O_2_	hydro-distillation	94310	(Yagi et al., 2013)
49	​	2-hydroxy-cyclopentadecanone	C_15_H_28_O_2_	hydro-distillation	543400	(Yagi et al., 2013)
50	​	(*11Z*)-hexadecenoic acid	C_16_H_30_O_2_	hydro-distillation	5312414	(Yagi et al., 2013)
51	​	(*15E*)-heptadecenal	C_17_H_32_O	hydro-distillation	5363097	(Yagi et al., 2013)
52	​	γ-nonalactone	C_9_H_16_O_2_	hydro-distillation	7710	(Yagi et al., 2013)
53	​	heptadecanoic acid	C_17_H_34_O_2_	MeOH-soluble fraction of EtOAc extract	10465	(Liu et al., 2015)
54	​	hexadecanol	C_16_H_34_O	hydro-distillation	2682	(Yagi et al., 2013)
55	​	linoleic acid	C_18_H_32_O_2_	hydro-distillation	528045	(Yagi et al., 2013)
56	​	linolenic acid	C_18_H_30_O_2_	hydro-distillation	5280934	(Yagi et al., 2013)
57	​	palmitic acid	C_16_H_32_O_2_	hydro-distillation	985	(Yagi et al., 2013)
58	​	tetradecanol	C_14_H_30_O	hydro-distillation	8209	(Yagi et al., 2013)
59	Volatile oil	1-octen-3-ol	C_8_H_16_O	hydro-distillation	18827	(Yagi et al., 2013)
60	​	1-octen-3-one	C_8_H_14_O	hydro-distillation	61346	(Yagi et al., 2013)
61	​	1,3-dihydrobenzofuran	C_8_H_8_O	hydro-distillation	10329	(Yagi et al., 2013)
62	​	2,3-octanedione	C_8_H_14_O_2_	hydro-distillation	11449	(Yagi et al., 2013)
63	​	2-cyclohexene-1-one	C_6_H_8_O	hydro-distillation	13594	(Yagi et al., 2013)
64	​	2-decanone	C_10_H_20_O	hydro-distillation	41092	(Yagi et al., 2013)
65	​	2-ethyl-1-hexanol	C_8_H_18_O	hydro-distillation	7720	(Yagi et al., 2013)
66	​	2-heptanone	C_7_H_14_O	hydro-distillation	8051	(Yagi et al., 2013)
67	​	2-hexylthiophene	C_10_H_16_S	hydro-distillation	87793	(Yagi et al., 2013)
68	​	2-pentylfuran	C_9_H_14_O	hydro-distillation	19602	(Yagi et al., 2013)
69	​	2-undecanone	C_11_H_22_O	hydro-distillation	87793	(Yagi et al., 2013)
70	​	3-octene-2-one	C_8_H_14_O	hydro-distillation	5363229	(Yagi et al., 2013)
71	​	3-ethyl-2,5-dimethylpyrazine	C_8_H_12_N_2_	hydro-distillation	25916	(Yagi et al., 2013)
72	​	3-methylthiopropanal	C_4_H_8_OS	hydro-distillation	18635	(Yagi et al., 2013)
73	​	6-methyl-5-hepten-2-one	C_8_H_14_O	hydro-distillation	9862	(Yagi et al., 2013)
74	​	4-keto-isophorone	C_9_H_12_O_2_	hydro-distillation	62374	(Yagi et al., 2013)
75	​	4-vinyl-*o*-guaiacol	C_9_H_10_O_2_	hydro-distillation	87793	(Yagi et al., 2013)
76	​	5-methyl-2,3,4,4-tetramethyl-cyclopent-2-enone	C_9_H_14_O	hydro-distillation	41092	(Yagi et al., 2013)
77	​	(*2E*)-heptenal	C_7_H_12_O	hydro-distillation	5283316	(Yagi et al., 2013)
78	​	(*2E*)-hexenal	C_6_H_10_O	hydro-distillation	5281168	(Yagi et al., 2013)
79	​	(*2E*)-nonen-4-one	C_9_H_16_O	hydro-distillation	448996	(Yagi et al., 2013)
80	​	(*2E*)-nonenal	C_9_H_16_O	hydro-distillation	5283335	(Yagi et al., 2013)
81	​	(*2E*)-octen-1-ol	C_11_H_22_O_2_	hydro-distillation	129881756	(Yagi et al., 2013)
82	​	(*2E*)-octenal	C_8_H_14_O	hydro-distillation	5283324	(Yagi et al., 2013)
83	​	(*2E,4E*)-nonadienal	C_9_H_14_O	hydro-distillation	496074471	(Yagi et al., 2013)
84	​	(*2E,6Z*)-nonadienal	C_9_H_14_O	hydro-distillation	643731	(Yagi et al., 2013)
85	​	benzaldehyde	C_7_H_6_O	hydro-distillation	240	(Yagi et al., 2013)
86	​	butyl methyl ether	C_5_H_12_O	hydro-distillation	12338	(Yagi et al., 2013)
87	​	decanal	C_10_H_20_O	hydro-distillation	8175	(Yagi et al., 2013)
88	​	furfural	C_5_H_4_O_2_	hydro-distillation	7362	(Yagi et al., 2013)
89	​	geraniol	C_10_H_18_O	hydro-distillation	637566	(Yagi et al., 2013)
90	​	heptanal	C_7_H_14_O	hydro-distillation	8130	(Yagi et al., 2013)
91	​	hexanal	C_6_H_12_O	hydro-distillation	6184	(Yagi et al., 2013)
92	​	hexanol	C_6_H_14_O	hydro-distillation	8103	(Yagi et al., 2013)
93	​	isophorone	C_9_H_14_O	hydro-distillation	6544	(Yagi et al., 2013)
94	​	methyl salicylate	C_8_H_8_O_3_	hydro-distillation	4133	(Yagi et al., 2013)
95	​	methylpyrazine	C_5_H_6_N_2_	hydro-distillation	7976	(Yagi et al., 2013)
96	​	nonanal	C_9_H_18_O	hydro-distillation	31289	(Yagi et al., 2013)
97	​	nonanol	C_9_H_20_O	hydro-distillation	8914	(Yagi et al., 2013)
98	​	octanal	C_8_H_16_O	hydro-distillation	454	(Yagi et al., 2013)
99	​	*o*-guaiacol	C_7_H_8_O_2_	hydro-distillation	460	(Yagi et al., 2013)
100	​	pentanol	C_5_H_12_O	hydro-distillation	6276	(Yagi et al., 2013)
101	​	phenylacetaldehyde	C_8_H_8_O	hydro-distillation	998	(Yagi et al., 2013)
102	​	phenylethanol	C_8_H_10_O	hydro-distillation	7409	(Yagi et al., 2013)
103	​	tetramethylpyrazine	C_8_H_12_N_2_	hydro-distillation	14296	(Yagi et al., 2013)
104	​	*trans*-2-(2-pentenyl)furan	C_9_H_12_O	hydro-distillation	5369957	(Yagi et al., 2013)
105	​	vinyl caproate	C_8_H_14_O_2_	hydro-distillation	76451	(Yagi et al., 2013)
106	​	γ-palmitolactone	C_16_H_30_O_2_	hydro-distillation	97747	(Yagi et al., 2013)

From a translational perspective, these chemically identified constituents should be interpreted in a graded manner, because only a subset currently has sufficient biological evidence to support mechanistic or therapeutic prioritization. Recent advances in high-throughput and multi-omics technologies have provided deeper insights into the chemical composition and biosynthesis of isoflavonoids and related metabolites in Pueraria species. For instance, integrated metabolomic and transcriptomic analyses have elucidated how key metabolites such as puerarin and daidzin accumulate at different developmental stages and correlate with the expression of UDP-glycosyltransferase and other biosynthetic genes, providing a comprehensive view of the regulatory networks governing secondary metabolite production (Hu et al., 2023).

Additionally, metabolome profiling combined with co-expression network analysis has been used to identify gene modules associated with flavonoid accumulation in distinct plant organs, leveraging PCA and WGCNA approaches to map regulatory landscapes (Zhu et al., 2024). These omics approaches exemplify the application of modern analytical methods such as liquid chromatography–tandem mass spectrometry (LC-MS/MS), PCA, and RNA sequencing (RNA-seq) to dissect the molecular mechanisms of bioactive compound synthesis and accumulation, thereby enriching phytochemical and pharmacological research on Pueraria. A separate integrated transcriptomic and targeted metabolomics study has further extended omics analysis to endogenous hormone dynamics during tuberous root expansion, illustrating the broader utility of high-throughput technologies in functional plant research (Wang et al., 2024).

### 4.1 Benzopyranoids

Among the benzopyranoids identified in *P. mirifica*, miroestrol and deoxymiroestrol are the most potent and diagnostic metabolites (Chansakaow et al., 2000a). Modern phytochemical evidence clarifies that deoxymiroestrol is the native constituent, whereas miroestrol is an oxidative artifact formed during post-harvest processing; crucially, deoxymiroestrol exhibits estrogenic potency an order of magnitude higher than its oxidized counterpart (Chansakaow et al., 2000). From a biosynthetic perspective, it is proposed that miroestrol originates from daidzein through a multi-step enzymatic sequence involving CYP81E-catalyzed hydroxylation, isoflavone reductase (IFR)-mediated reduction, and prenyltransferase (PT)-dependent prenylation. This pathway is substantiated by the significant co-expression of these candidate genes with daidzein accumulation and the corresponding phytochemical profiles of the species (Suntichaikamolkul et al., 2019).

However, the inherent chemical instability of these compounds poses a substantial challenge for product standardization, as improper storage can drastically diminish therapeutic efficacy (Udomsin et al., 2015). To address these limitations, research has bifurcated into two primary directions: Biotechnological Production: Optimized callus and cell suspension cultures have been developed, where the use of fructose as a carbon source can enhance deoxymiroestrol accumulation by up to 15.8-fold compared to wild roots (Udomsuk et al., 2011). Furthermore, complex synthetic routes, such as microwave-assisted siloxy-Cope rearrangement, have been established to provide high-purity crystalline standards for pharmacological validation (Ito et al., 2009; Shimokawa et al., 2013). Field-deployable Analysis: The field is shifting from laboratory-bound LC-MS/MS (Lee et al., 2017) toward high-throughput, point-of-need tools. Advanced immunochromatographic assays (ICA), immunochromatographic strips (ICS) (Krittanai et al., 2018), Fragment antigen-binding (Fab) fragments (Juengsanguanpornsuk et al., 2023), and ELISA techniques (Yusakul et al., 2018; Pongkitwitoon et al., 2019) are now employed to ensure supply chain integrity and verify metabolite concentrations (Chaingam et al., 2019; Sae-Foo et al., 2021; Masada et al., 2021; Chuphol et al., 2023).

### 4.2 Isoflavonoids and glycosides

Isoflavonoids are the most abundant phytoestrogens in *P. mirifica*, including daidzein, genistein, puerarin, and the species-specific marker kwakhurin. The species-specific marker kwakhurin is essential for authenticating the plant and distinguishing it from adulterants. Due to its extremely low natural abundance (0.0008%–0.007% w/w), developing robust synthesis and detection systems is crucial for quality control (Iwasaki et al., 2004; Chanpokapaiboon et al., 2018; Yusakul et al., 2019; Thanonkeo et al., 2024). Chemical synthesis of kwakhurin has evolved from early triisopropyl-protected routes to a total synthesis using MEM-protection (12% yield) (Ito et al., 2005), and recently to a highly efficient 3-step pathway (57% yield) validated by qNMR (Tsuji et al., 2020). For rapid standardization, immunodiagnostic methods offer significant advantages (Sakamoto et al., 2020; Phaisan et al., 2021; Sakamoto et al., 2022). A critical pharmacological consideration is that these metabolites predominantly exist as glycosides (e.g., daidzin). Their bioactivity depends on hydrolysis into aglycones by gut microbiota, a process subject to significant inter-individual variability (Makkliang et al., 2021). Recent omics findings (transcriptomic and proteomic) have elucidated the biosynthetic pathways, identifying key enzymes such as CHS, IFS, and UGT (Suntichaikamolkul et al., 2023). The expression of these enzymes is heavily influenced by the plant’s age and environmental factors, providing a scientific basis for the traditional emphasis on specific harvesting sites and maturity.

### 4.3 Coumestans

The coumestan profile, including coumestrol and unique mirificoumestans, further enriches the plant’s chemical diversity (Ingham et al., 1988). While their estrogenic activity is secondary to benzopyranoids, coumestrol exhibits multi-target effects, including anti-inflammatory and antioxidant activities. However, most current research remains at the stage of isolation and identification; direct pharmacological evaluation of these specific fractions from *P. mirifica* remains a priority for future studies.

### 4.4 Steroids, volatile oils, and fatty acids

Beyond the primary phytoestrogens, *P. mirifica* contains a complex matrix of secondary metabolites, including phytosterols (e.g., β-sitosterol, α-spinasterol), polysaccharides (Li et al., 2024), triterpenoids (squalene), and a diverse volatile profile comprising aldehydes, ketones, and terpenoids (e.g., germacrene D). Although these constituents are not considered diagnostic markers, metabolites like α-spinasterol exhibit documented cytotoxic and estrogen-like activities, potentially contributing to a synergistic “entourage effect” that modulates the plant’s overall therapeutic index (Jeon et al., 2005). Furthermore, the volatile oil fraction, while currently underutilized as cosmetic additives, presents an overlooked area for pharmacological research. From a quality control perspective, characterizing this “chemical background” is essential for ensuring batch-to-batch consistency and identifying interactions that may influence the bioavailability of major isoflavonoids. Future studies should transition from mere identification to physiological evaluation of these minor metabolites to support the rigorous standardization of *P. mirifica*-based functional products.

## 5 Pharmacological effects

**FIGURE 7:**
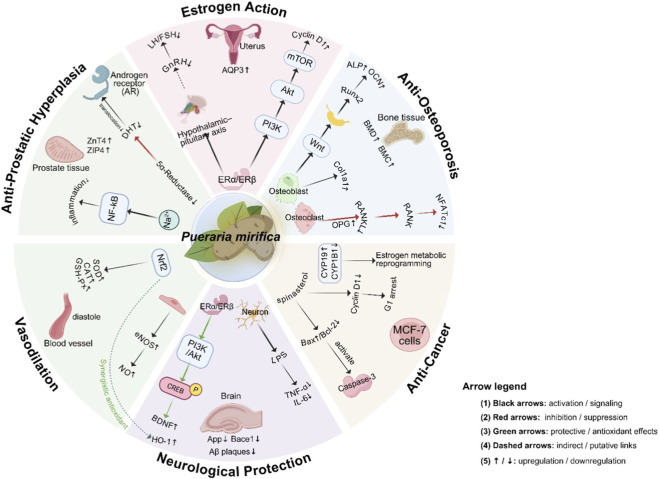
Multi-target pharmacological mechanisms of *Pueraria mirifica*. This diagram summarizes the major pharmacological effects and target systems. Arrows indicate activation, inhibition, or regulatory relationships. Arrow colors represent different types of regulatory relationships, as indicated in the legend (Created with BioRender.com).

The pharmacological literature on *P. mirifica* is extensive but uneven in evidentiary strength. The strongest body of support currently derives from phytochemical characterization, cell-based mechanistic studies, and animal experiments, whereas direct clinical validation remains comparatively limited. Accordingly, the biological effects discussed below should not be interpreted as equally established. In several areas, mechanistic plausibility is supported primarily by *in vitro* findings; in others, efficacy has been demonstrated in animal models but has not yet been confirmed in well-designed human studies (Jones et al., 1961; Ashby et al., 1999; Tiyasatkulkovit et al., 2012; Dickerson and Gore, 2007). Therefore, a critical distinction must be maintained between preclinical promise and clinically actionable evidence when evaluating the therapeutic potential of *P. mirifica*. The reported pharmacological effects of *P. mirifica*, together with their proposed mechanistic pathways and biological targets, are summarized in Table 3 and further illustrated in Figure 7. The pharmacological studies summarized in Table 3 include estrogenic, anti-osteoporotic, antioxidant, neuroprotective, vascular, reproductive, and anticancer-related evidence reported in previous *in vitro* and *in vivo* models (Bunmanop et al., 2011; Cherdshewasart and Sriwatcharakul, 2008a; Cherdshewasart and Sutjit, 2008c; Cherdshewasart et al., 2007; Geller and Studee, 2006; Hajirahimkhan et al., 2013; Islam et al., 2020; Jearapong et al., 2014; Jungsukcharoen et al., 2014; Kitisripanya et al., 2017a; Kongkaew et al., 2018; Lee et al., 2002; Liangdeng et al., 2024; Masrudin and Mohamad, 2015; Rattanapisit et al., 2021; Saetae, 2024; Singh, 2025; Trisomboon et al., 2006; Udomsuk et al., 2007; Udomsuk et al., 2011b; Xu et al., 2017; Yusakul et al., 2013).

**TABLE 3:** Reported pharmacological effects and proposed mechanisms of *P. mirifica*. This table summarizes pharmacological activities of isolated constituents and crude extracts of *P. mirifica* across *in vitro* and *in vivo* models, organized by test material, pharmacological activity, experimental model, major findings, and references.

Test material	Pharmacological activity	Experimental model	Major findings	Reference(s)
Deoxymiroestrol and miroestrol	Estrogenic activity	Male C57BL/6 mice	Reduction in intratesticular estradiol levels	(Udomsuk et al., 2011a)
Deoxymiroestrol and miroestrol	Estrogenic and antioxidant activities	C57BL/6 mice	Increased uterine weight and volume; reduced brain TBARS levels; decreased malondialdehyde (MDA) production; inhibition of lipid peroxidation	(Udomsuk et al., 2012; 2012b)
Miroestrol	Estrogenic activity	Ovariectomized (OVX) adult rats; immature female rats; post-mating female rats; adult male rats	Induction of vaginal epithelial cornification; inhibition of pituitary function; interference with pregnancy	(Jones & Pope, 1961)
Miroestrol	Neuroprotective activity	Ovariectomized (OVX) mice	Improvement of ovariectomy-induced spatial and non-spatial memory impairment; significant reduction in hippocampal and frontal cortical TBARS levels	(Monthakantirat et al., 2014)
Miroestrol	Antioxidant activity	Ovariectomized (OVX) ICR mice	Increased total and reduced GSH levels in the liver and uterus; elevated GSH/GSSG ratio; restoration of GPx, SOD, and CAT activity	(Chatuphonprasert et al., 2013)
Miroestrol	Antioxidant and antiproliferative activities	Female β-naphthoflavone (BNF)-induced ICR mice	Enhanced SOD and CAT activity in the liver and uterus; increased GSH levels and GSH/GSSG ratio; reduced uterine lipid peroxidation (MDA levels)	(Jearapong et al., 2014; Chatuphonprasert et al., 2016)
Puerarin	Estrogenic activity	Immature ovariectomized (OVX) Wistar rats; adult female Wistar rats with normal estrous cycles	Increased number of uterine glands and uterine cavity area; increased percentage of vaginal cornified cells after long-term administration	(Malaivijitnond et al., 2010)
Puerarin	Estrogenic and neuroprotective activities	Ovariectomized (OVX) Sprague-Dawley rats	Increased plasma growth hormone (GH) levels; increased trend in prolactin (PRL); reduced serum luteinizing hormone (LH) levels	(Loutchanwoot et al., 2016; Anukulthanakorn et al., 2016)
Puerarin	Antioxidant activity	Female Xanthium sibiricum (XSS) poisoning model in Sprague-Dawley rats	Reduced serum AST, ALT, ALP, LDH, BUN, and creatinine levels; modulation of MDA, GSH, and SOD levels	(Alkaç et al., 2022)
Puerarin	Antioxidant activity	Male streptozotocin (STZ)-induced diabetic Sprague-Dawley rats	Lowered blood glucose levels; alleviated kidney tissue damage	(Shen et al., 2009)
Puerarin	Antidepressant-like activity	Ovariectomized (OVX) ICR mice	Improved depression-like behavior, reduced immobility in FST and TST, increased hippocampal neurogenesis (DCX+ cells), lowered serum corticosterone, and partially restored uterine atrophy	(Tantipongpiradet et al., 2019)
Puerarin	Anti-inflammatory activity	Male high-fat diet (HFD)-induced MASLD model C57BL/6J mice	Reduced TC and LDL-C levels; increased HDL-C levels; alleviated hepatic fat deposition and inflammatory infiltration; repaired the intestinal barrier; regulated gut microbiota; increased short-chain fatty acid (SCFA) levels; inhibited systemic inflammation	(Sun et al., 2025)
Puerarin	Antioxidant activity	Sows of different parities	Increased GSH-Px and total antioxidant capacity (T-AOC) in serum and milk; reduced MDA levels; increased IL-10/IFN-γ ratio; enhanced IgA and IgG levels; improved offspring growth	(Cao et al., 2024)
Puerarin	Neuroprotective activity	Primary cultured mouse embryonic (E15-16) cortical neurons	Inhibition of Aβ1–42-induced neuronal death; reduced neuronal loss; promoted neurite growth; enhanced ER-dependent transcriptional activity; improved cell viability	(Li et al., 2017)
Puerarin	Anti-osteoporotic activity	Human osteoblast-like MG63 cells; human breast epithelial HBL-100 cells	Upregulation of OPG and downregulation of RANKL and IL-6 expression	(Wang et al., 2014; Wang et al., 2013)
Puerarin	Antiproliferative activity	Human breast cancer cell lines MCF-7 and MDA-MB-231	Inhibition of LPS-induced migration, invasion, and adhesion; reduced TNF-α and IL-6 secretion	(Liu et al., 2017)
Puerarin	Antiproliferative activity	Human colon cancer cell line HT-29	Dose-dependent inhibition of HT-29 cell proliferation and induction of apoptosis	(Yu and Li, 2006)
Puerarin	Anti-osteoporotic activity	Primary osteoblasts from cynomolgus monkeys	Promotion of osteoblast proliferation; upregulation of ALP and type I collagen mRNA; reduced RANKL/OPG ratio	(Tiyasatkulkovit et al., 2014)
Daidzein	Anti-inflammatory activity	LPS-induced acute lung injury model Sprague-Dawley rats; A549 cell line	Reduced lung wet-to-dry ratio, inflammatory cell infiltration in BALF, total protein concentration, MPO activity, and TNF-α/IL-6 levels	(Feng et al., 2015)
Daidzein	Antioxidant activity	Paclitaxel (PTX)-induced neuropathic pain model male BALB/c mice	Relief of mechanical, cold, and thermal hyperalgesia; improved histopathological damage; reduced oxidative stress and DNA damage; inhibition of neuronal apoptosis (decreased caspase-3 and Bax; increased Bcl-2); reduced IL-1β and TNF-α levels	(Zafar et al., 2023)
Daidzein	Anti-inflammatory activity	LPS-induced mouse mastitis model; mouse mammary epithelial cells (mMECs)	Alleviated mammary tissue damage; inhibited MPO activity; reduced IL-6 and IL-1β levels	(Cai et al., 2024)
Daidzein	Neuroprotective activity	Scopolamine-induced memory impairment model male ICR mice	Reversal of scopolamine-induced memory impairment	(Kim et al., 2010)
Daidzein	Anti-osteoporotic activity	Primary osteoblasts from cynomolgus monkeys	Promotion of osteoblast proliferation and upregulation of ALP and type I collagen mRNA	(Tiyasatkulkovit et al., 2014)
Daidzein	Anti-inflammatory activity	RAW264.7 mouse macrophages; human MDMs	Enhanced macrophage phagocytosis of apoptotic cells (efferocytosis)	(Yen et al., 2014)
Daidzein	Toxicological effects	Female Wistar rats	Upregulation of ERα, ERβ, and VEGF; interference with estrous cycle regulation	(Elsayed et al., 2022)
*P. mirifica* powder	Toxicological effects	Female Sprague-Dawley rats	Significantly reduced LH and FSH levels; prolonged estrus; induced mammary lobular hyperplasia; no stimulation of uterine or vaginal growth; reduced TC, LDL-C, and HDL-C levels; slightly increased triglycerides	(Chansakaow et al., 2000; Srasri et al., 2022)
*P. mirifica* powder	Estrogenic and anti-osteoporotic activities	Ovariectomized Sprague-Dawley rats	Promotion of vaginal and urethral cell maturation; improved bladder pressure and urethral closure; dose-dependent prevention of cancellous bone loss in the tibia, femur, and L4 vertebrae	(Manonai et al., 2009; Urasopon et al., 2008; Suthon et al., 2016; Trisomboon et al., 2006)
*P. mirifica* powder	Anti-osteoporotic activity	Orchidectomized (ORX) bone loss model Sprague-Dawley rats	Prevention of declines in BMD and BMC in cancellous and cortical bone	(Urasopon et al., 2007)
*P. mirifica* powder	Antiproliferative activity	Female 7,12-DMBA-induced breast cancer Sprague-Dawley rats	Significant reduction in breast tumor incidence, number, volume, and weight; prolonged latency; improved survival	(Cherdshewasart et al., 2007)
*P. mirifica* powder	Lipid-modulating activity	Male Wistar rats	Significantly reduced TC and LDL-C levels; improved AST, ALT, and ALP levels under high-cholesterol diet conditions; reduced HDL-C levels	(Charoenkul et al., 2005)
*P. mirifica* powder	Estrogenic activity	Immature ovariectomized (OVX) Wistar rats; adult female Wistar rats	3.6–4.6-fold increase in uterine weight; increased endometrial and myometrial area; induced vaginal cornification	(Malaivijitnond et al., 2010)
*P. mirifica* powder	Estrogenic activity	Ovariectomized (OVX) Wistar rats; orchidectomized (ORX) Wistar rats	Inhibition of LH and FSH in females; induced uterine weight gain and vaginal cornification; increased epididymal weight in males	(Malaivijitnond et al., 2004)
*P. mirifica* powder	Estrogenic activity	Ovariectomized (OVX) Wistar rats	Induction of vaginal cornification; increased uterine weight; inhibition of LH and FSH; improvement of menopausal symptoms; biphasic effects on MCF-7 cells (stimulatory at low dose and inhibitory at high dose)	(Malaivijitnond et al., 2006; Cherdshewasart et al., 2007; Cherdshewasart et al., 2008b; Anukulthanakorn et al., 2014)
*P. mirifica* powder	Toxicological effects	Male ICR mice	High-dose administration (100 mg/kg) decreased epididymal and seminal vesicle weight and reduced sperm motility	(Jaroenporn et al., 2007)
*P. mirifica* powder	Toxicological effects	Female ICR mice	High-dose administration induced persistent estrus, endometrial hyperplasia, and reduced mating and pregnancy rates	(Jaroenporn et al., 2007)
*P. mirifica* powder	Estrogenic activity	Adult female ICR mice; ovariectomized (OVX) rats; macaques	Inhibition of FSH and LH; induction of vaginal cornification and endometrial hyperplasia; reversible inhibition of fertility	(Jaroenporn et al., 2007)
*P. mirifica* powder	Antioxidant activity	Male rabbits with a high-fat diet (HFD)-induced atherosclerosis	Lowered plasma LDL levels; enhanced antioxidant capacity; improved vascular endothelial function; inhibited plaque formation	(Ratanachamnong et al., 2020)
*P. mirifica* powder	Vasodilatory activity	Ovariectomized (OVX) New Zealand White rabbits	Enhanced acetylcholine-induced vasodilation and improved vascular function	(Wattanapitayakul et al., 2005)
*P. mirifica* powder	Estrogenic and toxicological effects	Female cynomolgus monkeys (Macaca fascicularis)	High-dose administration prolonged or arrested the menstrual cycle and significantly reduced FSH, LH, estradiol, progesterone, and inhibin levels	(Trisomboon et al., 2004; 2004a; 2007)
*P. mirifica* powder	Anti-osteoporotic activity	Aged naturally menopausal cynomolgus monkeys	Attenuation of cortical bone BMD and BMC loss; improved bone geometry	(Kittivanichkul et al., 2016)
*P. mirifica* water extract	Estrogenic activity	Immature female Wistar rats; MCF-7 cells; ovariectomized (OVX) mice	Promotion of uterine growth (uterotrophic effect); stimulation of MCF-7 cell proliferation; promotion of vaginal opening and cornification	(Sookvanichsilp et al., 2008; Siangcham et al., 2010)
*P. mirifica* water extract	Genotoxic and estrogenic effects	Male Wistar rats	Significant increase in micronucleus frequency at 600–800 mg/kg	(Cherdshewasart et al., 2009)
*P. mirifica* extract	Neuroprotective activity	Ovariectomized (OVX) ICR mice	Improved spatial and non-spatial memory; alleviated uterine atrophy; improved SOD and CAT activity; reduced inflammatory mRNA expression	(Chulikhit et al., 2021)
*P. mirifica* extract	Estrogenic activity	Ovariectomized (OVX) rats; ovariectomized (OVX) monkeys	Increases uterine weight; promotes mammary bud proliferation; induces vaginal cornification	(Lin et al., 2017)
*P. mirifica* extract	Toxicological effects	Female Donryu rats (Crlj: DON)	Long-term administration promoted DMBA-induced breast cancer; increased uterine atypical hyperplasia and inflammation; altered ALP and BUN levels	(Kakehashi et al., 2016)
*P. mirifica* extract	Antioxidant activity	Human dermal fibroblasts (BJ cells)	Promoted fibroblast proliferation; protected against H2O2-induced oxidative damage; potential anti-aging effect	(Rani et al., 2021)
*P. mirifica* ethanol extract	Antidepressant-like activity	Ovariectomized (OVX) ICR mice	Shortened immobility time in the FST and TST; improved depression-like behavior; increased density of TPH-immunoreactive neurons in the dorsal raphe nucleus	(Tadsaichol et al., 2024)
*P. mirifica* ethanol extract	Neuroprotective activity	Castration-induced androgen-deficient Sprague-Dawley rats	Improved spatial learning and memory; restored synaptic plasticity	(Fainanta et al., 2022)
*P. mirifica* ethanol extract	Anti-osteoporotic activity	Orchidectomized rats; aged male SH rats; androgen-deficient cynomolgus macaques	Attenuated androgen deficiency-induced bone loss, particularly in cancellous and cortical bone	(Saetae et al., 2025)
*P. mirifica* ethanol extract	Estrogenic, neuroprotective, and anti-osteoporotic activities	Immature rats; MCF-7 cells	Improved depression-like behavior; restored synaptic plasticity and memory; protected against bone loss; promoted uterine growth and MCF-7 cell proliferation	(Sookvanichsilp et al., 2008)
*P. mirifica* ethanol extract	Estrogenic activity	Postmenopausal cynomolgus monkeys	Promotes vaginal epithelial maturation; lowers vaginal pH; improves vaginal atrophy	(Jaroenporn et al., 2014)
*P. mirifica* ethanol extract	Insecticidal activity	Aedes aegypti and Culex quinquefasciatus larvae	Larvicidal activity and insect growth regulatory effects	(Lapcharoen et al., 2005)
*P. mirifica* ethanol extract	Anti-osteoporotic activity	UMR106 rat osteoblast-like cells	Inhibition of cell proliferation, consistent with enhanced differentiation	(Tiyasatkulkovit et al., 2012)
*P. mirifica* ethanol extract	Antimutagenic activity	Salmonella typhimurium and Bacillus subtilis strains	Inhibition of AF-2- and benzo-α-pyrene-induced mutations	(Cherdshewasart et al., 2008)
*P. mirifica* ethyl acetate extract	Antioxidant activity	Ovariectomized (OVX) ICR mice	Increases GSH levels; restores GPx, SOD, and CAT activity	(Chatuphonprasert et al., 2013)
*P. mirifica* ethyl acetate extract	Anti-inflammatory activity	LPS-stimulated HAPI rat microglial cells	Inhibition of nitric oxide production, iNOS expression, and pro-inflammatory cytokines (MCP-1, IL-6, and TNF-α)	(Jantaratnotai et al., 2022)
*P. mirifica* ethyl acetate extract	Neuroprotective activity	Primary hippocampal neurons from embryonic day 19 Sprague-Dawley rats	Increased synaptophysin expression	(Chindewa et al., 2008)
*P. mirifica* ethyl acetate extract	Toxicological effects	Boar spermatozoa	Reduced sperm motility and spontaneous acrosome reaction	(Gray et al., 2015)
*P. mirifica* ethyl acetate extract	Neuroprotective activity	HT22 mouse hippocampal neurons	Scavenging of DPPH radicals and ROS; reduced glutamate-induced oxidative stress	(Prasansuklab et al., 2020)
*P. mirifica* ethyl acetate extract	Anti-osteoporotic activity	Ovariectomized (OVX) ICR mice	Prevents ovariectomy-induced bone loss	(Udomsuk et al., 2012)
*P. mirifica* ethyl acetate extract	Melanogenic activity	B16F10 melanoma cells; DU-145 cells	Significant induction of melanin production and enhanced tyrosinase activity	(Manosroi et al., 2018)
Coumestrol	Estrogenic activity	Juvenile female Alpk: AP rats	Induction of uterine, cervical, and vaginal weight gain; triggered cell proliferation and hyperplasia	(Ashby et al., 1999)
Dihydrotestosterone	Neuroprotective activity	Androgen-deficient Sprague-Dawley rats	Improved spatial learning and memory; restored synaptic plasticity	(Fainanta et al., 2022)
Spinasterol	Antiproliferative activity	Various cancer cell lines (breast, cervical, ovarian, colon, liver)	Antiproliferative effects across multiple cancer cell lines	(Jeon et al., 2005)

### 5.1 Estrogenic activities and mechanisms

*Pueraria mirifica* has a distinctive estrogenic profile among botanical phytoestrogens. Soy (*Glycine max*) and red clover (*Trifolium pratense*) are also important sources of phytoestrogens, but their compositions differ: soy mainly contains genistein and daidzein, whereas red clover is richer in biochanin A and formononetin in addition to these isoflavones (Mikulić et al., 2024; Messina et al., 2025). Current evidence indicates that these isoflavones bind both ERα and ERβ, often with a preference for ERβ, and are therefore better described as SERM-like, tissue-dependent ligands rather than simply weak ER agonists (Messina et al., 2025; Intharuksa et al., 2025; Viscardi et al., 2025). In contrast, *P. mirifica* also contains highly estrogenic chromenes, particularly miroestrol and deoxymiroestrol, which contribute to its notable estrogenic activity and warrant careful dose selection and safety evaluation (Juengsanguanpornsuk et al., 2021a). Accordingly, its pharmacological effects should be interpreted in a tissue-specific and dose-dependent manner. Endometrial stimulation and reproductive endocrine effects remain important considerations in estrogen-sensitive tissues, but the safety of phytoestrogens cannot be generalized because it depends on the botanical source, chemical composition, formulation, route of administration, and duration of use. Thus, the appropriate dose or concentration of *P. mirifica* should be selected according to the intended therapeutic purpose, particularly in older women undergoing menopausal management. Detailed clinical dosage considerations are discussed separately in the clinical applications section below (Viscardi et al., 2025).

In the reproductive system, *P. mirifica* metabolites directly stimulate target tissues such as the uterus and vagina, inducing epithelial keratinization and increasing uterine weight in a manner analogous to 17β-estradiol (E_2_) (Siangcham et al., 2010; Jaroenporn et al., 2014). However, this activity is characterized by a dual regulatory nature: while short-term or low-dose application can alleviate postmenopausal vaginal atrophy, chronic high-dose administration carries the risk of stimulating endometrial hyperplasia and potential oncogenesis (Cherdshewasart, 2003; Cherdshewasart et al., 2008a).

Beyond direct peripheral effects, *P. mirifica* modulates the hypothalamic–pituitary–gonadal (HPG) axis through negative feedback. By acting on the central nervous system, active metabolites inhibit the secretion of gonadotropin-releasing hormone (GnRH), subsequently suppressing the release of follicle-stimulating hormone (FSH) and luteinizing hormone (LH) (Saenphet et al., 2005; Loutchanwoot et al., 2016; Loutchanwoot and Vortherms, 2018). This mechanism is evidenced by the inhibition of compensatory gonadotropin rises in ovariectomized animal models and the disruption of ovulation or induction of amenorrhea in non-human primates (Cherdshewasart et al., 2004; Trisomboon et al., 2004).

At the cellular level, *P. mirifica* exhibits a complex biphasic biphasic dose-dependent pattern. For instance, in MCF-7 breast cancer cell lines, low concentrations (approximately 1 µg/mL) promote proliferation *via* the classical ERα pathway, whereas high concentrations (100–1000 µg/mL) shift toward anti-proliferative or cytotoxic effects (Chivapat et al., 2000; Juengsanguanpornsuk et al., 2021). The clinical efficacy is further dictated by biotransformation: most constituents exist as glycosides that require hydrolysis by intestinal microbiota into active aglycones (e.g., daidzein to equol) for effective absorption (Dickerson and Gore, 2007; Yusakul et al., 2016). Furthermore, individual differences in hepatic cytochrome P450 (CYP) enzyme polymorphisms significantly influence the metabolic activation or inactivation of these metabolites, leading to divergent clinical responses (Trisomboon et al., 2007; Suntichaikamolkul et al., 2023).

### 5.2 Anti-osteoporotic activity

**FIGURE 8:**
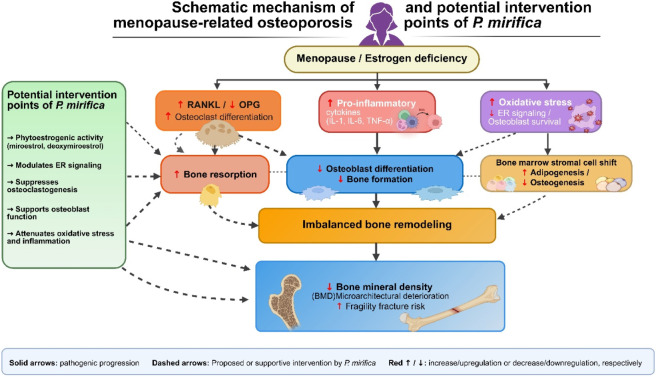
Schematic mechanism of menopause-related osteoporosis and the potential intervention points of *Pueraria mirifica*. Menopause-associated estrogen deficiency promotes osteoporosis through multiple interconnected pathways, including dysregulation of the OPG/RANKL axis, increased pro-inflammatory cytokine production, elevated oxidative stress, impaired estrogen receptor signaling, reduced osteoblast differentiation and survival, and a shift of bone marrow stromal cells toward adipogenesis rather than osteogenesis. These changes collectively lead to imbalanced bone remodeling, progressive loss of bone mineral density, microarchitectural deterioration, and increased fragility fracture risk. Based on current evidence, *Pueraria mirifica* may exert protective effects through phytoestrogenic activity and by modulating estrogen receptor signaling, osteoclastogenesis, osteoblast function, oxidative stress, and inflammation. Solid arrows indicate pathogenic progression, whereas dashed arrows indicate proposed or supportive intervention by *Pueraria mirifica*. Red upward and downward symbols (↑/↓) indicate increase/upregulation and decrease/downregulation, respectively (Created with BioRender.com).

Osteoporosis is closely associated with the decline in sex hormone levels, particularly estrogen deficiency, which disrupts bone remodeling homeostasis and shifts bone turnover toward excessive resorption (Cheng et al., 2022; Walker and Shane, 2023). Current evidence further indicates that postmenopausal osteoporosis involves dysregulation of the OPG/RANKL axis, enhanced osteoclastogenesis, altered osteoimmune interactions, chronic low-grade inflammation, oxidative stress, and increased bone marrow adiposity (Fischer and Haffner-Luntzer, 2022; Iantomasi et al., 2023; Niu et al., 2024; Luo et al., 2025). Within this mechanistic framework, *P. mirifica* and its active metabolites have demonstrated considerable anti-osteoporotic potential across various experimental models through complex multi-target regulation (Wiriyaampaiwong et al., 2012). The major mechanisms underlying menopause-related osteoporosis and the potential intervention points of *P. mirifica* are summarized in Figure 8.

The anti-osteoporotic effects of *P. mirifica* have been extensively validated in animal models simulating postmenopausal or androgen-deficient states. In both ovariectomized (OVX) female rats and orchidectomized (ORX) male rats, oral administration of *P. mirifica* extract dose-dependently prevents declines in bone mineral density (BMD) and bone mineral content (BMC) while improving bone microstructure (Urasopon et al., 2007; Urasopon et al., 2008a; Kittivanichkul et al., 2016; Suthon et al., 2016; Saetae, 2024; Saetae et al., 2025), with significant protective effects typically observed at doses of 100 mg/kg/day and above (Chatuphonprasert et al., 2013). However, in OVX female rats, a high dose (e.g., 1000 mg/kg) still induced significant uterine weight gain, indicating that its estrogen-like stimulatory effect on the female reproductive system persists (Wang et al., 2013). These anti-bone-loss effects appear to arise from bidirectional regulation of both bone formation and bone resorption processes.

Consistent with these *in vivo* findings, *in vitro* studies have shown that *P. mirifica* extract and puerarin promote osteoblast proliferation and differentiation, accompanied by upregulation of osteogenic marker genes such as alkaline phosphatase (ALP) and type I collagen (Col I) (Tiyasatkulkovit et al., 2012; Udomsuk et al., 2012a; Tiyasatkulkovit et al., 2014; Wang et al., 2014). A key molecular basis for these effects involves modulation of the OPG/RANKL system, a pivotal signaling axis in bone metabolism. By activating estrogen receptors, these metabolites increase osteoprotegerin (OPG) expression while reducing receptor activator of nuclear factor kappa-B ligand (RANKL) expression, thereby elevating the OPG/RANKL ratio and suppressing osteoclastogenesis and osteoclast function (Anukulthanakorn et al., 2014). This regulatory process appears to depend on ERα and ERβ activation and involves downstream signaling pathways such as MEK/ERK and PI3K/Akt (Monthakantirat et al., 2014; Kulczyński et al., 2021).

Despite these promising findings, several limitations remain. Most preclinical studies have relied on crude extracts rather than standardized fractions, making it difficult to determine which specific metabolite, such as puerarin or miroestrol, contributes most to the bone-sparing effect. In addition, although the OPG/RANKL axis has been well characterized *in vitro*, the lack of long-term safety data in ORX/OVX models limits current understanding of potential chronic systemic risks. The clinical translation of *P. mirifica* also remains challenging because the oral bioavailability of its active metabolites is generally low, and interindividual responses may vary considerably depending on gut microbiota composition, genetic polymorphisms of hepatic metabolic enzymes, and baseline hormone status (Chatuphonprasert et al., 2016). Taken together, current evidence supports the anti-osteoporotic potential of *P. mirifica*, but further studies using standardized preparations, long-term safety evaluation, and clinically relevant pharmacokinetic strategies are still needed.

### 5.3 Neuroprotective effects and cognitive enhancement

*Pueraria mirifica* and its metabolites have demonstrated neuroprotective potential across various preclinical models, primarily mediated through estrogen receptor (ER) signaling, antioxidant defense, and anti-inflammatory pathways (Prasansuklab et al., 2020; Singh, 2025). The neuroprotective efficacy appears highly state-dependent, typically manifesting in estrogen-deficient environments.

Phytoestrogens from *P. mirifica*, such as miroestrol and deoxymiroestrol, can cross the blood-brain barrier to modulate ERα and ERβ signaling. Unlike synthetic estrogens, certain metabolites exhibit a higher binding affinity for ERβ, which is critical for hippocampal-dependent memory and avoidance of ERα-mediated peripheral proliferation (Malaivijitnond et al., 2004). Activation of these receptors triggers downstream cascades, including the PI3K/Akt and MAPK/ERK pathways, subsequently upregulating neurotrophic factors such as BDNF and CREB, and synaptic markers like synaptophysin (Chindewa et al., 2008; Monthakantirat et al., 2014; Li et al., 2017). For instance, in ovariectomized (OVX) models, miroestrol significantly ameliorated spatial memory deficits, while genistein reversed chemically-induced impairment (Kim et al., 2010). However, it must be noted that these studies often utilize pure metabolites at concentrations exceeding those achievable through standard dietary intake of botanical drugs, raising questions regarding the translational relevance of such dosages.

*Pueraria mirifica* further mitigates neurodegeneration by downregulating APP and BACE1 expression and inhibiting tau hyperphosphorylation (Anukulthanakorn et al., 2016). While the upregulation of endogenous enzymes (SOD, CAT) and inhibition of NF-κB mediated neuroinflammation are well-documented (Sucontphunt et al., 2011), most current evidence relies on phenotypic observations in animal models. A critical gap remains in understanding the direct molecular targets—whether these botanical metabolites act as direct enzyme modulators or indirect gene regulators. Furthermore, while the selective activation of ERβ suggests a favorable safety profile regarding reproductive tissues, long-term clinical data on its neuroprotective safety margin in humans are still absent.

### 5.4 Antioxidant and anti-inflammatory effects

*Pueraria mirifica* possesses both significant antioxidant and phytoestrogen-like activities. These two effects often act synergistically, forming the basis for its efficacy in alleviating menopausal symptoms, providing cardiovascular protection, and offering neuroprotection (Trisomboon et al., 2006).

Its antioxidant mechanism is multi-dimensional. Beyond direct ROS scavenging (Ratanachamnong et al., 2020; Rani et al., 2021), the core antioxidant capacity of *P. mirifica* metabolites (notably puerarin and miroestrol) stems from the modulation of the Nrf2/ARE signaling pathway. Rather than acting as mere chemical scavengers, these metabolites function as “para-hormetic” stressors or direct ligands that trigger the nuclear translocation of Nrf2, thereby initiating the transcription of Phase II detoxifying enzymes, including HO-1, NQO1, and SOD (Cai et al., 2024; Cao et al., 2024). However, it is critical to distinguish between these molecular regulatory effects and phenotypic outcomes; while increased SOD/CAT levels are widely reported (Udomsuk et al., 2012; Udomsuk et al., 2012b; Jearapong et al., 2014; Laddha and Kulkarni, 2021; Alkaç et al., 2022), the precise upstream triggers (e.g., whether *via* ER-dependent or ER-independent kinase activation) remain partially elucidated in botanical drug research.

The anti-inflammatory repertoire of *P. mirifica* is characterized by its specificity to different pathological models. In acute states, daidzein suppresses the TLR4/MyD88/NF-κB axis and inhibits MAPK/AKT phosphorylation, effectively limiting the “cytokine storm” (Yen et al., 2014; Feng et al., 2015). A novel dimension of its efficacy is the regulation of the “gut-liver-brain axis” by puerarin, which enhances the intestinal barrier and promotes production of short-chain fatty acids (SCFAs) (Sun et al., 2025). Critically, while many studies emphasize the inhibition of iNOS and pro-inflammatory cytokines (IL-1β, TNF-α) (Jantaratnotai et al., 2022), few have addressed the potential “pro-inflammatory” risks of *P. mirifica* at supra-physiological doses where estrogenic overstimulation might occur.

The convergence of these pathways is evident in estrogen-deficiency pathologies. Activation of ERβ by *P. mirifica* isoflavonoids often leads to the trans-repression of inflammatory genes (Jaroenporn et al., 2006; Atiq et al., 2019), suggesting that its anti-inflammatory benefits are inextricably linked to its receptor affinity (Zafar et al., 2023).

### 5.5 Complex effects on tumor cells: the phytoestrogen paradox

*Pueraria mirifica* and its active metabolites exhibit a complex “duality” in their effects on tumors, particularly hormone-sensitive ones. This biphasic effect is intrinsically linked to their structural similarity to 17β-estradiol and their dose-dependent affinity for estrogen receptors (ERα and ERβ) (Trisomboon et al., 2007). Under specific conditions, *P. mirifica* metabolites demonstrate significant anti-tumor potential. In various breast, ovarian, and colorectal cancer cell lines, these metabolites induce apoptosis by upregulating the Bax/Bcl-2 ratio and activating Caspase-3 (Yu and Li, 2006; Liu et al., 2017; Alizamir et al., 2025; Islam et al., 2025). Furthermore, they modulate carcinogen-metabolizing enzymes, specifically inhibiting the pro-carcinogenic CYP1B1 while upregulating protective phase II enzymes like NQO1 (Chatuphonprasert et al., 2016). Recent bioinformatics and molecular docking analyses suggested that *P. mirifica* may have anti-hepatocellular carcinoma (HCC) potential through targets including ADH1B and GHR (Li et al., 2025). Importantly, this prediction was not based solely on *in silico* evidence, as the same study further showed that the candidate compound hederagenin inhibited HepG2 cell viability and increased ADH1B protein expression, providing preliminary experimental support for ADH1B-related activity (Li et al., 2025). In addition, independent *in vitro* studies have shown that puerarin exerts anti-HCC effects by increasing chemosensitivity, inhibiting proliferation, and promoting apoptosis in liver cancer cells (Wu et al., 2017; Zhang et al., 2017). However, direct experimental validation of GHR as a functional target of *P. mirifica* in HCC remains limited and warrants further investigation.

A critical concern for clinical translation is the risk of tumor stimulation. Due to their potent estrogenic activity, *P. mirifica* extracts can act as ER agonists in low-estrogen environments or in ER-positive tumor contexts. *In vitro* studies have confirmed a classic “inverted U-shaped” dose-response curve: low concentrations of extract can promote MCF-7 cell proliferation, with inhibitory effects only occurring at significantly higher, possibly supra-physiological, doses (Cherdshewasart et al., 2007a).

The therapeutic window for the “anti-tumor” effect of *P. mirifica* is narrow and highly dependent on the host’s endogenous hormonal milieu and the specific metabolite profile (e.g., the ratio of miroestrol to deoxymiroestrol). The absence of standardized extract formulations in many reported studies (Jeon et al., 2005) complicates the comparison of results across different laboratories. For individuals with a history of hormone-dependent malignancies, the use of *P. mirifica* botanical drugs presents a non-negligible risk that warrants strict regulatory oversight and further longitudinal safety studies.

### 5.6 Effects on blood lipids and cardiovascular function

*Pueraria mirifica* and its active metabolites exhibit multifaceted regulatory effects on lipid metabolism and cardiovascular function. These actions are predominantly characterized as “estrogen-mimetic” cardioprotection, manifesting most effectively in hormone-deficient or hyperlipidemic environments (Cherdshewasart et al., 2008).

Preclinical evidence suggests that *P. mirifica* effectively lowers serum total cholesterol (TC) and LDL-C while improving the HDL-C/LDL-C ratio in high-cholesterol diet models (Charoenkul et al., 2005; Ratanachamnong et al., 2020). Mechanistically, this transition involves the activation of metabolic “sensing” pathways, including AMPK, PPARα, and FXR, which collectively coordinate hepatic lipid oxidation and cholesterol efflux (Ou et al., 2025). However, a critical assessment of the literature reveals significant heterogeneity in outcomes: while beneficial in pathological models, some studies report a transient elevation of triglycerides (TG) in subjects on a normal diet (Charoenkul et al., 2005). This discrepancy may stem from the non-selective activation of hepatic ERs, which can paradoxically stimulate VLDL synthesis, a nuanced risk often overlooked in purely descriptive reviews.

*Pueraria mirifica* enhances endothelium-dependent vasodilation, primarily by activating endothelial nitric oxide synthase (eNOS) and increasing nitric oxide (NO) bioavailability (Wattanapitayakul et al., 2005). It is important to note that most current findings rely on acute or sub-acute animal models. The efficacy of *P. mirifica* in preventing chronic cardiovascular events, such as myocardial infarction or stroke, remains unproven in large-scale clinical settings.

While its multi-target action offers a broader therapeutic window than isolated statins, its systemic estrogenic load remains a primary safety concern. The cardiovascular benefits must be meticulously weighed against the potential risks to hormone-sensitive tissues, necessitating further research into tissue-selective estrogen receptor modulators (SERMs) derived from *P. mirifica*.

### 5.7 Other pharmacological potentials

*Pueraria mirifica* exhibits diverse secondary pharmacological effects, ranging from systemic immunomodulation to tissue-specific protective roles.

Immunomodulatory and Prostatic Health: Crude polysaccharides from *P. mirifica* have shown non-specific immunostimulatory activity *in vitro* by promoting CD69 expression in T-lymphocytes (Burana-Osot et al., 2010). More specifically, in male reproductive health, aqueous extracts have demonstrated potential in attenuating testosterone-induced benign prostatic hyperplasia (BPH). This effect is mediated through the inhibition of 5α-reductase and the restoration of zinc ion homeostasis *via* zinc transporter upregulation (Masrudin and Mohamad, 2015; Mohamad et al., 2019). Critically, these findings suggest a potential role for *P. mirifica* in modulating male hormonal imbalances, though the interference with androgen signaling requires careful long-term safety monitoring.

Psychopharmacology and Dermal Applications: Recent studies (Tantipongpiradet et al., 2019; Tadsaichol et al., 2024) have highlighted antidepressant-like effects, where metabolites activate ERβ to upregulate BDNF and tryptophan hydroxylase (TPH), the rate-limiting enzyme in serotonin synthesis. These findings position *P. mirifica* as a candidate for managing postmenopausal depressive symptoms. In dermatology, *n*-hexane extracts were found to stimulate melanin synthesis *via* tyrosinase activation (Manosroi et al., 2018), offering a scientific basis for traditional hair-darkening claims.

Genoprotective and Agricultural Applications: Beyond human health, *P. mirifica* ethanol extracts exhibit significant antimutagenic potential by protecting against chemical-induced genetic damage (Lapcharoen et al., 2005). Additionally, its insect growth-regulating activity against *Aedes aegypti* larvae suggests potential for bio-pesticide development (Cherdshewasart et al., 2009). However, the dual use of the plant in both therapeutic and pesticidal contexts necessitates a rigorous evaluation of systemic toxicity to ensure that bioactive metabolites do not pose risks of genotoxicity in human consumers.

### 5.8 Pharmacokinetics, metabolism, and individual variability

The clinical efficacy of *P. mirifica* is significantly influenced by its bioavailability, metabolic pathways, and the substantial inter-individual variability in response (Dickerson and Gore, 2007). These factors are crucial for its pharmacological activity and need careful consideration to optimize therapeutic use.

1. Bioavailability: The oral bioavailability of puerarin, one of the key active compounds in *P. mirifica*, remains a major challenge. In rodent models, its bioavailability is reported to be approximately 7%, and it is even lower in non-human primates (less than 1%) (Anukunwithaya et al., 2018). This limited absorption is attributed to the rigid C-glycosidic structure of puerarin, which resists intestinal hydrolysis, and the active efflux *via* P-glycoprotein (P-gp) transporters, compounded by significant first-pass glucuronidation by uridine 5′-diphospho-glucuronosyltransferase enzymes (UGTs) (Namken et al., 2021). These pharmacokinetic limitations result in low systemic concentrations, though puerarin tends to accumulate in tissues such as the hippocampus and bone matrix after chronic dosing. This suggests that steady-state tissue concentrations might be more relevant for efficacy than acute plasma peaks.
2. Metabolism and Interindividual Variability: One of the most critical factors in individual responsiveness is the gut microbiota-mediated conversion of daidzein into S-equol, a metabolite with significantly higher estrogenic potency and stronger affinity for ERβ. However, only 30%–50% of the human population possesses the necessary anaerobic bacteria for this biotransformation (Boonchird et al., 2010), leading to varying clinical outcomes. Additionally, genetic polymorphisms in CYP450 enzymes (such as CYP1A2 and CYP1B1) create a unique “metabolic fingerprint” for each individual, which complicates the establishment of a universal therapeutic window. This inter-individual variability in metabolism can result in differences in the therapeutic and adverse effects of *P. mirifica.*
3. Phytoestrogen Responsiveness: The variable conversion of daidzein into S-equol underscores the importance of considering phytoestrogen responsiveness when assessing *P. mirifica*‘s clinical effects. Individuals who are poor converters of daidzein may derive suboptimal benefits, despite receiving standard dosages. This variability is essential to consider when evaluating therapeutic responses, as it significantly influences the estrogenic effects of the plant. Further studies are needed to better understand the relationship between individual metabolic profiles and therapeutic outcomes, enabling more personalized treatment strategies.
4. Risk of Drug Interactions: *P. mirifica* extracts can modulate hepatic enzymes, particularly inhibiting CYP3A4 and CYP1A2, which poses a risk for interactions with co-administered medications, including hormonal therapies and drugs with narrow therapeutic indices (Kitisripanya et al., 2018). These interactions may alter the pharmacokinetics of other drugs, potentially leading to adverse effects or reduced efficacy. Therefore, future research must prioritize standardized pharmacokinetic studies in humans to transition from empirical dosing to precision-based botanical drug administration.

### 5.9 Toxicological profile and safety margin

The potent phytoestrogenic activity of *P. mirifica* is the foundation of its pharmacological effects, but it also constitutes its primary source of safety risk. Current research indicates that its toxicity is clearly dose-dependent and time-cumulative, suggesting that the safety margin for long-term use may be relatively narrow (Cherdshewasart, 2003).

Major toxic manifestations are concentrated in the reproductive, hematological, and potential carcinogenic systems. In female rodent models, high-dose exposure (≥100 mg/kg/day) results in significant endocrine-disrupting effects, including endometrial hyperplasia and hydrometra. Critically, in non-human primates, these dosages can induce irreversible amenorrhea, raising concerns about its long-term impact on the hypothalamic-pituitary-ovarian (HPO) axis (Chivapat et al., 2000; Trisomboon et al., 2005; Jaroenporn et al., 2007). In males, reproductive toxicity is manifested as reduced sperm motility and testicular atrophy (Jaroenporn et al., 2006). Beyond reproductive organs, hematopoietic toxicity—including bone marrow suppression and non-reversible hemolysis—has been observed in chronic toxicity studies, highlighting a systemic risk that persists even after cessation of treatment (Chivapat et al., 2000).

While the acute oral LD_50_ remains low, the Lowest Observed Adverse Effect Level (LOAEL) is approximately 100 mg/kg/day in rats. The pharmacological dosages used in many efficacy studies (10–50 mg/kg) are in close proximity to this LOAEL, indicating a narrow safety window. The therapeutic doses often used in pharmacological studies (10–50 mg/kg) fall within a range that is dangerously close to the LOAEL, underscoring the fragility of the safety margin. Much of the existing safety data relies on rodent models, which may fail to accurately predict human risk due to interspecies variations in hepatic CYP450 isoforms and gut microbiota-mediated metabolism of deoxymiroestrol. Furthermore, the lack of standardized metabolic profiling in these studies complicates the differentiation between therapeutic efficacy and potential carcinogenicity, particularly concerning estrogen-sensitive mammary tumors (Kakehashi et al., 2016). In summary, while *P. mirifica* exhibits significant pharmacological benefits, the gap between therapeutic and toxic doses suggests that careful consideration of the safety margins is necessary. Further research involving large-scale human trials is crucial to better define these boundaries and ensure the safe use of the plant for therapeutic purposes.

## 6 Clinical applications

Clinical evidence suggests that *P. mirifica* may have therapeutic relevance for menopausal symptom management and genitourinary health, particularly in relation to vasomotor symptoms, vaginal atrophy, and selected quality-of-life outcomes. However, the translational strength of this evidence remains limited because most studies have involved relatively small sample sizes, short intervention periods, heterogeneous formulations, and incomplete standardization of dose and constituent composition. In addition, long-term safety assessment remains insufficient, particularly with regard to endometrial effects, endocrine regulation, and the use of *P. mirifica* in women with differing baseline hormonal status or comorbidities. For these reasons, the current human data should be interpreted cautiously, with attention to both the reported benefits and the important limitations that affect translational reliability. The published human clinical studies evaluating Pueraria mirifica formulations in menopausal symptom management and genitourinary health are summarized in Table 4.

**TABLE 4:** Clinical studies of *P. mirifica* in menopausal symptom management and genitourinary health. This table summarizes published human clinical studies of *P. mirifica* formulations, including study design, dosage, main outcomes, and reported adverse events.

Dosage and formulation	Sample size, n (completed)	Duration, months	Primary endpoint(s)	Main clinical findings	Reported adverse events	Reference(s)
Oral raw powder capsules, 200 mg/day or every other day	5 (4 completed)	12	Improvement in menopausal symptoms (subjective reports)	Significant improvement in menopausal symptoms; firmer skin; improved sleep; decreased cholesterol	Mild weight gain (0.5–3 kg)	(Muangman and Cherdshewasart, 2001)
Oral extract capsules, 50 or 100 mg/day	37 (18 completed)	6	Modified Greene Climacteric Scale (MGCS)	Greater improvement in MGCS score at 100 mg/day; fluctuating increase in serum estradiol (peak 114.2 pg/mL); stable FSH and LH; slight decrease in lipoproteins	Transient anemia, elevated liver enzymes, headache, palpitations, and insomnia; 1 withdrawal	(Lamlertkittikul and Chandeying, 2004)
Oral root powder capsules, 50 or 100 mg/day	10 (8 completed)	6	MGCS; hot flash and night sweat score	Fluctuating increase in serum estradiol (peak 114.2 pg/mL); stable FSH and LH; slight decrease in lipoproteins	Transient increase in creatinine and BUN; 2 withdrawals due to hypertension and heavy head sensation	(Chandeying and Lamlertkittikul, 2007)
Oral raw powder, 50 mg/day	74 (60 completed)	6	Change in MGCS	Significant reduction in MGCS score; laboratory indices generally stable	Elevated triglycerides in some patients (>400 mg/dL)	(Chandeying and Lamlertkittikul, 2007)
Oral extract capsules, 20/30/50 mg/day	86 (71 completed)	6	Vaginal dryness and dyspareunia scores; Vaginal Health Index (VHI); vaginal pH; vaginal cytology (MI, MV); bone-specific alkaline phosphatase (BAP); lipid profile	Improved maturation index; increased VHI; decreased vaginal pH; reduced dryness and dyspareunia scores; decreased BAP; slight increase in triglycerides within the normal range	Mild bloating and headache; no difference from placebo	(Manonai et al., 2007, 2008)
Oral raw powder capsules, 20/30/50 mg/day	86 (71 completed)	6	Lipid profile; bone-specific alkaline phosphatase (BAP); endometrial thickness and histology; safety	Significant decrease in BAP; no change in endometrial thickness or histology	Mild elevation in triglycerides in both groups	(Manonai et al., 2008)
Oral dried root powder, 100 mg/day	19	2	Lipid profile (HDL-C, LDL-C, apo A1, apo B)	Increased HDL-C by 34%; decreased LDL-C by 17%; increased apo A1 by 40%; decreased apo B by 9%	No serious adverse events reported	(Okamura et al., 2008)
Oral extract tablets, 25 or 50 mg/day	52 (all completed)	6	MGCS	Significant reduction in MGCS score in both groups; slightly faster improvement in musculoskeletal symptoms at 50 mg/day	Sporadic mild symptoms, including bloating and headache	(Virojchaiwong et al., 2011)
Vaginal gel (6% extract), 0.5 g/dose	82 (41 per group)	4	Vaginal symptom score; Vaginal Health Index (VHI); Vaginal Maturation Index (VMI); Lactobacillus grading; endometrial thickness	Significant improvement in VMI, symptom scores, and VHI; restoration of dominant Lactobacillus flora	No serious adverse events; no abnormal bleeding	(Suwanvesh et al., 2017)
Vaginal gel (5%), 0.5 g/dose	60 (30 per group)	4	Nugent score; Vaginal Health Index (VHI)	Relief of dyspareunia and vaginal dryness; significant improvement in Nugent score and VHI	No serious adverse events; well tolerated	(Pitsouni et al., 2018)
Vaginal gel (5%), 0.5 g/dose	60 (30 per group)	3	Nugent score; vaginal pH; fungal culture; Vaginal Health Index (VHI); genitourinary syndrome of menopause questionnaire; endometrial thickness	Significant improvement in Nugent score and VHI	No serious adverse events; well tolerated	(Sritonchai et al., 2020)

### 6.1 Improvement of vasomotor symptoms and general menopausal symptoms

Clinical investigations have consistently highlighted the potential of *P. mirifica* in alleviating vasomotor symptoms (VMS), which are primarily attributed to its high-potency chromenes, such as deoxymiroestrol. This metabolite exhibits significantly higher estrogenic activity compared to common soy isoflavones by interacting more robustly with estrogen receptors (ERα and ERβ) to stabilize hypothalamic thermoregulation (Li Y. et al., 2025).

Early pilot studies utilized daily doses of 200 mg of raw root powder, reporting marked subjective improvements in the Modified Greene Climacteric Scale (MGCS) (Muangman and Cherdshewasart, 2001). However, these open-label designs are inherently susceptible to significant placebo effects, necessitating more rigorous validation. A subsequent Phase III randomized controlled trial (n = 60) compared a daily dose of 50 mg of *P. mirifica* against standard conjugated equine estrogen (CEE) therapy, finding no significant difference in efficacy for reducing MGCS scores over 24 weeks (Chandeying and Sangthawan, 2007). This suggests that *P. mirifica* might serve as a viable alternative for patients seeking non-hormonal options, although its dose-response relationship remains poorly defined. These studies generally reported a significant decline in symptom scores after 3–6 months of treatment (Lamlertkittikul and Chandeying, 2004; Chandeying and Lamlertkittikul, 2007). Further dose-comparison studies (n = 52) indicated that 25 mg and 50 mg doses were equivalently effective (Virojchaiwong et al., 2011), hinting at a potential “ceiling effect” in its therapeutic window.

Critically, while the initial efficacy is promising, the clinical evidence remains fragmented. Most trials suffer from small cohort sizes (n < 71), short follow-up durations (typically <6 months), and a lack of standardized phytochemical characterization of the extracts used. Without quantification of the highly unstable deoxymiroestrol in the trial medication, the reproducibility of these clinical outcomes across different batches remains questionable.

### 6.2 Improvement of genitourinary syndrome of menopause (GSM)

Topical administration of *P. mirifica* represents a specialized therapeutic modality for Genitourinary Syndrome of Menopause (GSM), specifically addressing vaginal atrophy, dryness, and dyspareunia. Multiple randomized controlled trials (RCTs) have demonstrated that a 12-week regimen of 5%–6% *P. mirifica* vaginal gel (standardized for isoflavonoid content) significantly elevates the Vaginal Health Index (VHI) and Vaginal Maturation Index (VMI) (Manonai et al., 2007; Sritonchai et al., 2019). Unlike systemic hormone replacement therapy (HRT), local application facilitates a rapid restoration of vaginal pH and improves the microenvironment, as evidenced by lower Nugent scores—a critical indicator for reduced bacterial vaginosis risk (Warinsiriruk et al., 2022).

From a hemodynamic perspective, Doppler ultrasound assessments have confirmed that *P. mirifica* gel significantly enhances vaginal arterial perfusion, evidenced by a marked decrease in the Pulsatility Index (PI) and Resistance Index (RI) (Sritonchai et al., 2020). This vascular response is likely mediated by the activation of estrogen receptors on the vaginal vascular endothelium, promoting nitric oxide-dependent vasodilation. Notably, several comparative studies indicate that *P. mirifica* may outperform Conjugated Equine Estrogen (CEE) cream in alleviating dysuria, possibly due to a more balanced interaction with ERβ receptors prevalent in the lower urinary tract (Suwanvesh et al., 2017).

Critically, the localized safety profile is supported by evidence that short-term use (up to 24 weeks) does not induce endometrial hyperplasia or significant changes in serum estradiol levels. However, a significant research gap remains: the majority of current studies focus on “healthy” postmenopausal cohorts. Future investigations must prioritize safety data for high-risk populations, such as breast cancer survivors with contraindications for traditional HRT, to establish *P. mirifica* as a validated alternative in oncological supportive care.

### 6.3 Impact on lipid metabolism and skeletal integrity

Emerging evidence suggests that *P. mirifica* exerts systemic modulatory effects on metabolic and skeletal homeostasis, although the clinical magnitude remains debated. Regarding lipid profiles, a short-term study (n = 19) demonstrated that a 100 mg/day dose of *P. mirifica* powder significantly elevated HDL-C and Apolipoprotein A1, while concomitantly reducing LDL-C and Apolipoprotein B (Okamura et al., 2008). This lipid-lowering effect mimics the action of endogenous 17β-estradiol, likely through the upregulation of LDL receptors in the liver. However, it is noteworthy that some subjects exhibited an elevation in triglycerides (TG)—a common side effect of oral estrogenic compounds due to their impact on hepatic lipid synthesis—necessitating caution in patients with pre-existing hypertriglyceridemia.

In terms of bone health, *P. mirifica* appears to function as a natural Selective Estrogen Receptor Modulator (SERM). A randomized, double-blind, placebo-controlled trial (n = 71) indicated that doses of 20–50 mg/day significantly suppressed Bone-specific Alkaline Phosphatase (BAP) levels without stimulating endometrial thickening (Manonai et al., 2008). This suggests a potent anti-resorptive effect, potentially superior to that of soy-derived isoflavones due to the unique presence of deoxymiroestrol.

Critically, the current clinical landscape for these indications is significantly limited by “methodological heterogeneity.” The discrepancy in dosages across studies (20 mg vs. 100 mg) and the lack of long-term fracture-reduction data or cardiovascular endpoint studies (e.g.,.CIMT measurements) prevent a definitive recommendation. Furthermore, the variability in the phytochemical “fingerprint” of the root powders used in these trials makes it difficult to establish a standardized therapeutic window for metabolic health.

### 6.4 Safety profile, toxicological risks, and pharmacological interactions

Clinical data suggest that short-term (<12 months) administration of *P. mirifica* is generally well-tolerated, with adverse events (AEs) typically limited to transient anemia, headache, and bloating. However, its potent estrogenic activity necessitates a rigorous appraisal of potential systemic risks.

Beyond common AEs, rare but severe cardiac risks have been identified. A critical case report highlighted a patient with a KCNQ1-T587M mutation who developed life-threatening Torsades de Pointes (TdP) following *P. mirifica* supplementation (Kashiwa et al., 2021). This suggests that certain isoflavones or chromenes within the plant may interfere with cardiac I_Kr_ potassium channels, posing a specific risk for individuals with “silent” long QT syndrome.

A large-scale survey (n = 1,200) in Japan indicated that 16.2% of users experienced estrogen-related AEs, including abnormal vaginal bleeding and mastalgia (Chiba et al., 2022). Critically, these reports often involve unregulated dietary supplements where the concentration of deoxymiroestrol—a metabolite significantly more potent than 17β-estradiol—is frequently unstandardized. This highlights a major “quality control crisis” in the *P. mirifica* industry, where the lack of marker-based standardization leads to unpredictable biological responses.

Although clinical data remain limited, *in vitro* studies suggest that *P. mirifica* metabolites may modulate cytochrome P450 (CYP) enzymes, particularly CYP1A1, CYP1A2, and CYP1B1. This raises a theoretical concern for patients who are concurrently receiving medications with narrow therapeutic indices or hormonal therapies for breast cancer, such as tamoxifen, because metabolic interference could potentially compromise treatment efficacy or increase toxicity.

In summary, although *P. mirifica* represents a promising alternative for the management of vasomotor symptoms (VMS) and genitourinary syndrome of menopause (GSM), the current evidence remains limited by small sample sizes and the absence of long-term clinically meaningful endpoints, such as breast density changes or cardiovascular events. Future research should prioritize long-term oncogenic safety trials, rigorous pharmacokinetic characterization to define the therapeutic window, and mandatory standardization of deoxymiroestrol content in all clinical research materials.

## 7 Discussion

Taken together, the current literature supports *P. mirifica* as a botanically distinctive and pharmacologically promising phytoestrogenic species; however, the overall evidence base remains uneven. Phytochemical diversity and preclinical pharmacology are relatively well documented, whereas clinical validation, pharmacokinetic characterization, long-term safety assessment, and product standardization remain underdeveloped. This imbalance explains why the literature contains substantial mechanistic and experimental promise, yet still falls short of supporting fully standardized and clinically reliable application. A clearer translational framework is therefore required to connect ethnopharmacological relevance, prioritized bioactive constituents, mechanistic pathways, and human therapeutic use.

## 8 Limitations of the study

While this review provides valuable insights, several limitations should be acknowledged. First, the current body of clinical evidence on *P. mirifica* is largely derived from small-scale and short-duration studies, leaving important gaps in the understanding of long-term safety and efficacy. Second, substantial variability among commercial *P. mirifica* products, driven by differences in production processes and material standardization, may influence both clinical outcomes and safety profiles. Third, the absence of comprehensive safety monitoring data, particularly from post-marketing surveillance, complicates the establishment of a clear and reliable safety profile for *P. mirifica* in the general population.

## 9 Future recommendations

To address the major translational barriers identified in this review, future research should focus on several key priorities. First, standardization strategies should be strengthened through authenticated botanical identification, control of cultivation and harvest conditions, chemical fingerprinting with prioritized marker compounds, batch-to-batch consistency testing, and bioactivity-linked quality evaluation. Second, clinical research should move toward larger, randomized, double-blind, placebo-controlled studies using well-characterized formulations, clearly defined dosages, and extended follow-up periods to better establish efficacy and long-term safety. Third, pharmacokinetic and metabolic studies should be expanded to clarify oral bioavailability, metabolite transformation, microbiota-related variability, tissue distribution, and potential herb–drug interactions. Fourth, advanced methodologies, including metabolomics, transcriptomics, proteomics, and integrated multi-omics approaches, should be more explicitly incorporated into botanical drug development in order to identify key active constituents, define mechanistic pathways, support marker discovery, and improve mechanism-guided quality control. Finally, greater regulatory harmonization will be necessary to reduce the variability of commercial *P. mirifica* products and to facilitate more reproducible clinical translation.

## 10 Conclusion

In conclusion, *P. mirifica* is a botanically distinctive and pharmacologically important phytoestrogenic species with considerable potential for menopause-related applications and other estrogen-responsive conditions. However, the current evidence base remains uneven: phytochemical diversity and preclinical activities are relatively well documented, whereas clinical validation, pharmacokinetic characterization, long-term safety assessment, and formulation standardization remain insufficient. Future progress will depend not only on the generation of additional data, but also on improving the quality, consistency, and translational relevance of that evidence. In particular, priority should be given to phytochemical prioritization, stronger standardization frameworks, better characterization of metabolism and tissue selectivity, rigorous long-term safety evaluation, and clinically robust trials using well-defined products. Only through such an integrated and multidisciplinary approach can the therapeutic potential of *P. mirifica* be more reliably advanced toward evidence-based botanical drug development.

## Glossary

ALP: alkaline phosphatase
ARMS: amplification refractory mutation system
AST: aspartate aminotransferase
BACE1: β-site amyloid precursor protein cleaving enzyme 1
BAP: bone-specific alkaline phosphatase
BDNF: brain-derived neurotrophic factor
BMC: bone mineral content
BMD: bone mineral density
BPH: benign prostatic hyperplasia
BUN: blood urea nitrogen
CAT: catalase
CEE: conjugated equine estrogen
CHS: chalcone synthase
CYP: cytochrome P450
DCM: dichloromethane
**E**_**2**_: 17β-estradiol
ELISA: enzyme-linked immunosorbent assay
eNOS: endothelial nitric oxide synthase
ER: estrogen receptor
ERα: estrogen receptor alpha
ERβ: estrogen receptor beta
FSH: follicle-stimulating hormone
GBIF: Global Biodiversity Information Facility
GH: growth hormone
GnRH: gonadotropin-releasing hormone
GPx: glutathione peroxidase
GSH: glutathione
GSM: genitourinary syndrome of menopause
HFD: high-fat diet
HPG: hypothalamic–pituitary–gonadal
HPO: hypothalamic–pituitary–ovarian
HRT: hormone replacement therapy
ICA: immunochromatographic assay
ICS: immunochromatographic strip
IFS: isoflavone synthase
IL-6: interleukin-6
ITS: internal transcribed spacer
LC-MS/MS: liquid chromatography–tandem mass spectrometry
LDH: lactate dehydrogenase
LDL-C: low-density lipoprotein cholesterol
LH: luteinizing hormone
LOAEL: lowest observed adverse effect level
LPS: lipopolysaccharide
MASLD: metabolic dysfunction-associated steatotic liver disease
MEK/ERK: mitogen-activated protein kinase kinase/extracellular signal-regulated kinase
MGCS: Modified Greene Climacteric Scale
NADES: natural deep eutectic solvents
NF-κB: nuclear factor kappa-B
NMR: nuclear magnetic resonance
NO: nitric oxide
OPG: osteoprotegerin
ORX: orchidectomized
OVX: ovariectomized
PCA: principal component analysis
PCR-RFLP: polymerase chain reaction–restriction fragment length polymorphism
P-gp: P-glycoprotein
PI: pulsatility index
PI3K/Akt: phosphatidylinositol 3-kinase/protein kinase B
POWO: Plants of the World Online
PRL: prolactin
RANKL: receptor activator of nuclear factor kappa-B ligand
RI: resistance index
RNA-seq: RNA sequencing
SCFAs: short-chain fatty acids
SERM: selective estrogen receptor modulator
SOD: superoxide dismutase
TC: total cholesterol
TG: triglycerides
TPH: tryptophan hydroxylase
UGT: UDP-glycosyltransferase
UGT2: UDP-glycosyltransferase 2
UPLC-MS/MS: ultra-performance liquid chromatography–tandem mass spectrometry
VHI: Vaginal Health Index
VMI: Vaginal Maturation Index
VMS: vasomotor symptoms
WGCNA: weighted gene co-expression network analysis

## References

[B1] Alizamir A. Khanaghaei M. Mirzavi F. Nokhodchi A. Ghavami L. Taghizadeh B. (2025). Puerarin as a multifaceted anticancer agent: mechanisms, targets, and therapeutic potential across multiple cancers. Chin. Herb. Med. 10.1016/j.chmed.2025.11.001 PMC13390012 42488847

[B2] Alkaç Z. K. Korkak F. A. Dağoğlu G. Incili C. A. Hark B. D. Tanyıldızı S. (2022). Puerarin mitigates oxidative injuries, opening of mitochondrial permeability transition pores and pathological damage associated with liver and kidney in *Xanthium strumarium*-intoxicated rats. Toxicon 213, 13–22. 10.1016/j.toxicon.2022.04.004 35427636

[B3] Anukulthanakorn K. Jareonporn S. Malaivijitnond S. (2014). Simple, sensitive and reliable *in vivo* assays to evaluate the estrogenic activity of endocrine disruptors. Reprod. Med. Biol. 13, 37–45. 10.1007/s12522-013-0161-1 29699148 PMC5906850

[B4] Anukulthanakorn K. Parhar I. S. Jaroenporn S. Kitahashi T. Watanbe G. Malaivijitnond S. (2016). Neurotherapeutic effects of *Pueraria mirifica* extract in early- and late-stage cognitive impaired rats. Phytother. Res. 30, 929–939. 10.1002/ptr.5595 26915634

[B5] Anukunwithaya T. Poo P. Hunsakunachai N. Rodsiri R. Malaivijitnond S. Khemawoot P. (2018). Absolute oral bioavailability and disposition kinetics of puerarin in female rats. BMC Pharmacol. Toxicol. 19, 25. 10.1186/s40360-018-0216-3 29801513 PMC5970530

[B6] Ashby J. Tinwell H. Soames A. Foster J. (1999). Induction of hyperplasia and increased DNA content in the uterus of immature rats exposed to coumestrol. Environ. Health Perspect. 107, 819–822. 10.1289/ehp.99107819 10504149 PMC1566597

[B7] Atiq A. Shal B. Naveed M. Khan A. Ali J. Zeeshan S. (2019). Diadzein ameliorates 5-fluorouracil-induced intestinal mucositis by suppressing oxidative stress and inflammatory mediators in rodents. Eur. J. Pharmacol. 843, 292–306. 10.1016/j.ejphar.2018.12.014 30529194

[B8] Bang M.-H. Lee D.-G. Baek Y.-S. Cho J.-G. Han M.-W. Choi K.-S. (2013). A new miroestrol glycoside from the roots of *Pueraria mirifica* . Chem. Nat. Compd. 49, 443–445. 10.1007/s10600-013-0634-9

[B9] Boonchird C. Mahapanichkul T. Cherdshewasart W. (2010). Differential binding with ERalpha and ERbeta of the phytoestrogen-rich plant *Pueraria mirifica* . Braz. J. Med. Biol. Res. 43, 195–200. 10.1590/S0100-879X2009007500026 20027484

[B10] Boonsnongcheep P. Korsangruang S. Soonthornchareonnon N. Chintapakorn Y. Saralamp P. Prathanturarug S. (2010). Growth and isoflavonoid accumulation of *Pueraria candollei var. candollei* and *P. candollei var. mirifica* cell suspension cultures. Plant Cell Tissue Organ Cult. 101, 119–126. 10.1007/s11240-010-9668-x

[B11] Bunmanop S. Sakuanrungsirikul S. Manakasem Y. (2011). White Kwao Krua variety classification by botanical characteristics and ISSR-touchdown PCR technique. Russ. J. Genet. 47, 819–828. 21938956

[B210] Burana-Osot J. Pattanapanyasat K. Soonthornchareonnon N. Sukapirom K. Toida T. (2010). Characterisation and immuno-stimulating activity of polysaccharides from Thai medicinal plants. Nat. Prod. Res. 24, 1403–1412. 10.1080/14786410902940974 20812129

[B12] Cai X. Zhou Z. Kan X. Xu P. Guo W. Fu S. (2024). Daidzein relieves lipopolysaccharide-induced mastitis through inhibiting MAPKs and AKT/NF-κB p65 signaling pathways. Rev. Bras. Farmacogn. 34, 853–864. 10.1007/s43450-024-00529-4

[B13] Cain J. C. (1960). Miroestrol: an oestrogen from the plant *Pueraria mirifica* . Nature 188, 774–777. 10.1038/188774a0 13689829

[B14] Cao S. Li X. Yin H. Wang J. Liu J. (2024). Dietary puerarin supplementation improves immune response and antioxidant capacity of sows. Antioxidants 13, 290. 10.3390/antiox13030290 38539824 PMC10967452

[B15] Chaingam J. Kitisripanya T. Krittanai S. Sakamoto S. Tanaka H. Putalun W. (2019). Development of a simple and rapid method for the detection of isomiroestrol in *Pueraria candollei* by an immunochromatographic strip test. J. Nat. Med. 73, 577–583. 10.1007/s11418-019-01307-6 30976950

[B16] Chandeying V. Lamlertkittikul S. (2007a). Challenges in the conduct of Thai herbal scientific study: efficacy and safety of phytoestrogen, *Pueraria mirifica* (Kwao Keur Kao), phase I, in the alleviation of climacteric symptoms in perimenopausal women. J. Med. Assoc. Thai 90, 1274–1280. 17710964

[B17] Chandeying V. Sangthawan M. (2007). Efficacy comparison of *Pueraria mirifica* (PM) against conjugated equine estrogen (CEE) with/without medroxyprogesterone acetate (MPA) in the treatment of climacteric symptoms in perimenopausal women: phase III study. J. Med. Assoc. Thai 90, 1720–1726. 17957910

[B18] Chanpokapaiboon K. Khoonrit P. Yusakul G. Juengwatanatrakul T. Putalun W. Tanaka H. (2018). A recombinant Fab antibody against kwakhurin as a tool for sensitive indirect competitive ELISA. Curr. Pharm. Biotechnol. 19, 1170–1176. 10.2174/1389201020666181226105223 30585546

[B19] Chansakaow S. Ishikawa T. Seki H. Sekine K. Okada M. Chaichantipyuth C. (2000). Identification of deoxymiroestrol as the actual rejuvenating principle of “Kwao Keur,” *Pueraria mirifica*. The known miroestrol may be an artifact. J. Nat. Prod. 63, 173–175. 10.1021/np990547v 10691701

[B20] Chansakaow S. Ishikawa T. Sekine K. Okada M. Higuchi Y. Kudo M. (2000a). Isoflavonoids from *Pueraria mirifica* and their estrogenic activity. Planta Med. 66, 572–575. 10.1055/s-2000-8603 10985090

[B21] Charoenkul K. Phivthong-Ngam L. Srichairat S. (2005). Subchronic exposure of *Pueraria mirifica* in normal and high cholesterol diet-fed rats: influence on lipid profile and toxicity. Thai J. Pharmacol. 27, 67–75.

[B22] Charoensup W. Intharuksa A. Yanaso S. Khamnuan S. Chansakaow S. Sirisa-Ard P. (2024). Botanical biometrics: exploring morphological, palynological, and DNA barcoding variations in White Kwao Krua (*Pueraria candollei* Grah. ex Benth. and *P. mirifica* Airy Shaw and Suvat.). Horticulturae 10, 162.

[B23] Chatuphonprasert W. Udomsuk L. Monthakantirat O. Churikhit Y. Putalun W. Jarukamjorn K. (2013). Effects of *Pueraria mirifica* and miroestrol on the antioxidation-related enzymes in ovariectomized mice. J. Pharm. Pharmacol. 65, 447–456. 10.1111/jphp.12003 23356854

[B24] Chatuphonprasert W. Jarukamjorn K. Putalun W. (2016). Regulation of cancer-related genes—Cyp1a1, Cyp1b1, Cyp19, Nqo1 and Comt—expression in β-naphthoflavone-treated mice by miroestrol. J. Pharm. Pharmacol. 68, 475–484. 10.1111/jphp.12531 26893163

[B25] Cheng C. H. Chen L. R. Chen K. H. (2022). Osteoporosis due to hormone imbalance: an overview of the effects of estrogen deficiency and glucocorticoid overuse on bone turnover. Int. J. Mol. Sci. 23 (3), 1376. 10.3390/ijms23031376 35163300 PMC8836058

[B26] Cherdshewasart W. (2003). Toxicity tests of a phytoestrogen-rich herb, *Pueraria mirifica* . J. Sci. Res. Chula Univ. 28, 1–12.

[B27] Cherdshewasart W. Sriwatcharakul S. (2007). Major isoflavonoid contents of the 1-year-cultivated phytoestrogen-rich herb, *Pueraria mirifica* . Biosci. Biotechnol. Biochem. 71, 2527–2533. 10.1271/bbb.70316 17928711

[B28] Cherdshewasart W. Sriwatcharakul S. (2008). Metabolic activation promotes estrogenic activity of the phytoestrogen-rich plant. Maturitas 59, 128–136. 10.1016/j.maturitas.2008.01.002 18313242

[B29] Cherdshewasart W. Sutjit W. Pulcharoen K. (2008). Correlation of antioxidant activity and major isoflavonoid contents of the phytoestrogen-rich *Pueraria mirifica* and *Pueraria lobata* tubers. Phytomedicine 15, 38–43. 10.1016/j.phymed.2007.07.058 17890070

[B30] Cherdshewasart W. Cheewasopit W. Picha P. (2004). The differential anti-proliferation effect of white (*Pueraria mirifica*), red (*Butea superba*), and black (*Mucuna collettii*) Kwao Krua plants on the growth of MCF-7 cells. J. Ethnopharmacol. 93, 255–260. 10.1016/j.jep.2004.03.041 15234761

[B31] Cherdshewasart W. Kitsamai Y. Malaivijitnond S. (2007). Evaluation of the estrogenic activity of the wild *Pueraria mirifica* by vaginal cornification assay. J. Reprod. Dev. 53, 385–393. 10.1262/jrd.18065 17229996

[B32] Cherdshewasart W. Subtang S. Dahlan W. (2007). Major isoflavonoid contents of the phytoestrogen rich-herb *Pueraria mirifica* in comparison with *Pueraria lobata* . J. Pharm. Biomed. Anal. 43, 428–434. 10.1016/j.jpba.2006.07.013 16930918

[B33] Cherdshewasart W. Panriansaen R. Picha P. (2007a). Pretreatment with phytoestrogen-rich plant decreases breast tumor incidence and exhibits lower profile of mammary ERalpha and ERbeta. Maturitas 58, 174–181. 10.1016/j.maturitas.2007.08.001 17870258

[B34] Cherdshewasart W. Sriwatcharakul S. Malaivijitnond S. (2008). Variance of estrogenic activity of the phytoestrogen-rich plant. Maturitas 61, 350–357. 10.1016/j.maturitas.2008.09.017 18980816

[B35] Cherdshewasart W. Traisup V. Picha P. (2008b). Determination of the estrogenic activity of wild phytoestrogen-rich *Pueraria mirifica* by MCF-7 proliferation assay. J. Reprod. Dev. 54, 63–67. 10.1262/jrd.19002 18160771

[B36] Cherdshewasart W. Sutjit W. Pulcharoen K. Chulasiri M. (2009). The mutagenic and antimutagenic effects of the traditional phytoestrogen-rich herbs, *Pueraria mirifica* and *Pueraria lobata* . Braz. J. Med. Biol. Res. 42, 816–823. 10.1590/S0100-879X2009000900008 19738987

[B37] Chiba T. Tousen Y. Nishijima C. Umegaki K. (2022). The prevalence of dietary supplements that claim estrogen-like effects in Japanese women. Nutrients 14, 4509. 10.3390/nu14214509 36364772 PMC9653890

[B38] Chindewa R. Lapanantasin S. Sanvarinda Y. Chongthammakun S. (2008). *Pueraria mirifica*, phytoestrogen-induced change in synaptophysin expression *via* estrogen receptor in rat hippocampal neuron. J. Med. Assoc. Thai 91, 208–214. 18389986

[B39] Chivapat S. Chavalittumrong P. Rattanajarasroj S. Chuthaputti A. Panyamang S. (2000). Toxicity study of *Pueraria mirifica* Airy Shaw et Suvatabandhu. Bull. Med. Sci. 42, 202–223.

[B40] Cho J.-G. Park H.-J. Huh G.-W. Lee S. S. Bang M.-H. Choi K.-S. (2014). Flavonoids from *Pueraria mirifica* roots and quantitative analysis using HPLC. Food Sci. Biotechnol. 23, 1815–1820. 10.1007/s10068-014-0248-4

[B41] Chulikhit Y. Sukhano W. Daodee S. Putalun W. Wongpradit R. Khamphukdee C. (2021). Effects of *Pueraria candollei var. mirifica* (Airy Shaw and Suvat.) Niyomdham on ovariectomy-induced cognitive impairment and oxidative stress in the mouse brain. Molecules 26, 3442. 10.3390/molecules26113442 34198932 PMC8201258

[B42] Chuphol N. Nokkaew N. Makkliang F. Sae-Foo W. Phaisan S. Putalun W. (2023). Immunochromatographic assay for miroestrol and deoxymiroestrol, its cross-reactivity, and application in *Pueraria mirifica* (White Kwao Krua) analysis. Phytochem. Anal. 34, 421–430. 10.1002/pca.3223 36950953

[B43] Dickerson S. M. Gore A. C. (2007). Estrogenic environmental endocrine-disrupting chemical effects on reproductive neuroendocrine function and dysfunction across the life cycle. Rev. Endocr. Metab. Disord. 8, 143–159. 10.1007/s11154-007-9048-y 17674209

[B44] Elsayed D. H. Helmy S. A. Dessouki A. A. El-Nahla A. M. Abdelrazek H. M. A. El-Hak H. N. G. (2022). Influence of genistein and diadizine on regularity of estrous cycle in cyclic female Wistar rat: interaction with estradiol receptors and vascular endothelial growth factor. Open Vet. J. 12, 639–648. 10.5455/OVJ.2022.v12.i5.8 36589405 PMC9789761

[B45] Fainanta T. Jaroenporn S. Wititsuwankul P. Malaivijitnond S. (2022). Comparison of neuroprotective effects of dihydrotestosterone, 17β-estradiol, and *Pueraria mirifica* herb extract on cognitive impairment in androgen deficient male rats. Horm. Behav. 143, 105198. 10.1016/j.yhbeh.2022.105198 35609404

[B46] Feng G. Sun B. Li T.-Z. (2015). Daidzein attenuates lipopolysaccharide-induced acute lung injury *via* toll-like receptor 4/NF-kappaB pathway. Int. Immunopharmacol. 26, 392–400. 10.1016/j.intimp.2015.04.002 25887269

[B47] Fischer V. Haffner-Luntzer M. (2022). Interaction between bone and immune cells: implications for postmenopausal osteoporosis. Semin. Cell Dev. Biol. 123, 14–21. 10.1016/j.semcdb.2021.05.014 34024716

[B48] Geller S. E. Studee L. (2006). Soy and red clover for mid-life and aging. Climacteric 9, 245–263. 10.1080/13697130600736934 16857655 PMC1780039

[B49] Gray S. L. Lackey B. R. Boone W. R. (2015). Impact of kudzu and puerarin on sperm function. Reprod. Toxicol. 53, 54–62. 10.1016/j.reprotox.2015.03.010 25828059

[B50] Hajirahimkhan A. Dietz B. M. Bolton J. L. (2013). Botanical modulation of menopausal symptoms: mechanisms of action? Planta Med. 79, 538–553. 10.1055/s-0032-1328187 23408273 PMC3800090

[B51] Hu X. Zhu T. Min X. He J. Hou C. Liu X. (2023). Integrated metabolomic and transcriptomic analysis of puerarin biosynthesis in *Pueraria montana var. thomsonii* at different growth stages. Genes 14, 2230. 10.3390/genes14122230 38137052 PMC10742406

[B52] Huynh H. T. T. Cao T. T. T. Nguyen C. X. Nguyen D. T. Nguyen N. T. B. Cao D. T. T. (2024). Comparison in DNA barcoding, gene expression and chemical of *Pueraria mirifica* plants. J. Anim. Plant Sci. 34 (5), 1303–1316. 10.36899/JAPS.2024.5.0812

[B53] Iantomasi T. Romagnoli C. Palmini G. Donati S. Falsetti I. Miglietta F. (2023). Oxidative stress and inflammation in osteoporosis: molecular mechanisms involved and the relationship with microRNAs. Int. J. Mol. Sci. 24 (4), 3772. 10.3390/ijms24043772 36835184 PMC9963528

[B54] Ingham J. L. Tahara S. Dziedzic S. Z. (1986). A chemical investigation of *Pueraria mirifica* roots. Z. Naturforsch. C 41, 403–408. 10.1515/znc-1986-0406

[B55] Ingham J. L. Markham K. R. Dziedzic S. Z. Pope G. S. (1986a). Puerarin 6″-O-β-apiofuranoside, a C-glycosylisoflavone O-glycoside from *Pueraria mirifica* . Phytochemistry 25, 1772–1775. 10.1016/s0031-9422(00)81262-5

[B56] Ingham J. L. Tahara S. Dziedzic S. Z. (1988). Coumestans from the roots of *Pueraria mirifica* . Z. Naturforsch. C 43, 5–10. 10.1515/znc-1988-1-203

[B57] Ingham J. L. Tahara S. Dziedzic S. Z. (1989). Minor isoflavones from the roots of *Pueraria mirifica* . Z. Naturforsch. C 44, 724–726. 10.1515/znc-1989-9-1002

[B58] Intharuksa A. Kitamura M. Peerakam N. Charoensup W. Ando H. Sasaki Y. (2020). Evaluation of White Kwao Krua (*Pueraria candollei* Grah. ex Benth.) products sold in Thailand by molecular, chemical, and microscopic analyses. J. Nat. Med. 74, 106–118. 10.1007/s11418-019-01351-2 31377923

[B59] Intharuksa A. Arunotayanun W. Na Takuathung M. Chaichit S. Prasansuklab A. Chaikhong K. (2025). Daidzein and genistein: natural phytoestrogens with potential applications in hormone replacement therapy. Int. J. Mol. Sci. 26 (14), 6973. 10.3390/ijms26146973 40725220 PMC12294992

[B60] Islam M. T. Rahman M. T. Mia E. Kamli H. Hasan A. M. W. Uddin M. B. (2025). Anticancer potential of daidzin: a comprehensive literature review. Med. Oncol. 42, 285. 10.1007/s12032-025-02839-6 40569503

[B61] Ito F. Iwasaki M. Watanabe T. Ishikawa T. Higuchi Y. (2005). The first total synthesis of kwakhurin, a characteristic component of a rejuvenating plant, “Kwao Keur”: toward an efficient synthetic route to phytoestrogenic isoflavones. Org. Biomol. Chem. 3, 674–681. 10.1039/b414955f 15703807

[B62] Ito F. Kumamoto T. Yamaguchi K. Ishikawa T. (2009). Synthetic studies toward miroestrols: trials for elongation of the methyl group of 5-substituted 2-methyl-2-cyclohexanone to 3-methyl-2-butenyl function. Tetrahedron 65, 771–785. 10.1016/j.tet.2008.11.055

[B63] Iwasaki M. Watanabe T. Ishikawa T. Chansakaow S. Higuchi Y. Tahara S. (2004). Kwakhurin (I), a unique isoflavone with rejuvenating activity from Kwao Keur: further characterization by 2D-NMR spectrometry and synthesis of triisopropylkwakhurin. Heterocycles 63, 1375–1392. 10.3987/com-04-10060

[B64] Jantaratnotai N. Thampithak A. Utaisincharoen P. Pinthong D. Sanvarinda P. (2022). Inhibition of LPS-induced microglial activation by the ethyl acetate extract of *Pueraria mirifica* . Int. J. Environ. Res. Public Health 19, 12920. 10.3390/ijerph191912920 36232220 PMC9566591

[B65] Jaroenporn S. Malaivijitnond S. Wattanasirmkit K. Trisomboon H. Watanabe G. Taya K. (2006). Effects of *Pueraria mirifica*, an herb containing phytoestrogens, on reproductive organs and fertility of adult male mice. Endocrine 30, 93–101. 10.1385/ENDO:30:1:93 17185797

[B66] Jaroenporn S. Malaivijitnond S. Wattanasirmkit K. Watanabe G. Taya K. Cherdshewasart W. (2007). Assessment of fertility and reproductive toxicity in adult female mice after long-term exposure to *Pueraria mirifica* herb. J. Reprod. Dev. 53, 995–1005. 10.1262/jrd.18151 17585183

[B67] Jaroenporn S. Urasopon N. Watanabe G. Malaivijitnond S. (2014). Improvements of vaginal atrophy without systemic side effects after topical application of *Pueraria mirifica*, a phytoestrogen-rich herb, in postmenopausal cynomolgus macaques. J. Reprod. Dev. 60, 238–245. 10.1262/jrd.2013-144 24748397 PMC4085389

[B68] Jearapong N. Chatuphonprasert W. Jarukamjorn K. (2014). Miroestrol, a phytoestrogen from *Pueraria mirifica*, improves the antioxidation state in the livers and uteri of β-naphthoflavone-treated mice. J. Nat. Med. 68, 173–180. 10.1007/s11418-013-0788-6 23812874

[B69] Jeon G. C. Park M. S. Yoon D. Y. Shin C. H. Sin H. S. Um S. J. (2005). Antitumor activity of spinasterol isolated from Pueraria roots. Exp. Mol. Med. 37, 111–120. 10.1038/emm.2005.15 15886524

[B70] Jones H. E. Pope G. S. (1961). A method for the isolation of miroestrol from *Pueraria mirifica* . J. Endocrinol. 22, 303–312. 10.1677/joe.0.0220303 13790533

[B71] Jones H. E. Waynforth H. B. Pope G. S. (1961). The effect of miroestrol on vaginal cornification, pituitary function and pregnancy in the rat. J. Endocrinol. 22, 293–302. 10.1677/joe.0.0220293 13790534

[B72] Juengsanguanpornsuk W. Yusakul G. Kitisripanya T. Krittanai S. Juengwatanatrakul T. Sakamoto S. (2021). Quantification of methylisomiroestrol, a phytoestrogen of *Pueraria candollei*, by enzyme-linked immunosorbent assay in comparison with high-performance liquid chromatography. J. Pharm. Biomed. Anal. 192, 113674. 10.1016/j.jpba.2020.113674 33120305

[B73] Juengsanguanpornsuk W. Yusakul G. Kraithong W. Putalun W. (2021a). Simple preparation and analysis of a phytoestrogen-rich extract of *Pueraria candollei var. mirifica* and its *in vitro* estrogenic activity. J. Herb. Med. 29, 100463. 10.1016/j.hermed.2021.100463

[B74] Juengsanguanpornsuk W. Poopanee N. Krittanai S. Sakamoto S. Tanaka H. Putalun W. (2023). Immunoaffinity separation of miroestrol and deoxymiroestrol from *Pueraria candollei var. mirifica* (Airy Shaw and Suvat.) Niyomdham using fragment antigen-binding antibody produced *via Escherichia coli* . Phytochem. Anal. 34, 632–640. 10.1002/pca.3251 37254639

[B75] Jungsukcharoen J. Dhiani B. A. Cherdshewasart W. Vinayavekhin N. Sangvanich P. Boonchird C. (2014). *Pueraria mirifica* leaves, an alternative potential isoflavonoid source. Biosci. Biotechnol. Biochem. 78, 917–926. 10.1080/09168451.2014.910091 25036114

[B76] Jungsukcharoen J. Chokchaichamnankit D. Srisomsap C. Cherdshewasart W. Sangvanich P. (2016). Proteome analysis of *Pueraria mirifica* tubers collected in different seasons. Biosci. Biotechnol. Biochem. 80, 1070–1080. 10.1080/09168451.2016.1141035 26940377

[B77] Kakehashi A. Yoshida M. Tago Y. Ishii N. Okuno T. Gi M. (2016). *Pueraria mirifica* exerts estrogenic effects in the mammary gland and uterus and promotes mammary carcinogenesis in Donryu rats. Toxins 8, 275. 10.3390/toxins8110275 27827907 PMC5127102

[B78] Kandale P. Shikare V. Varhade P. Awachar A. Chopade R. Nagrik S. (2025). Hormone replacement therapy in menopause: evidence-based benefits, risks, and evolving practice guidelines. Int. J. Sci. R. Tech. 2 (5), 614–626. 10.5281/zenodo.15529463

[B79] Kashemsanta M. L. Suvatabandhu K. Airy Shaw H. A. (1952). A new species of Pueraria (Leguminosae) from Thailand, yielding an oestrogenic principle. Kew Bull. 7, 549–552. 10.2307/4117811

[B80] Kashiwa A. Hosaka Y. Takahashi K. Ohno S. Wada Y. Makiyama T. (2021). *Pueraria mirifica*, an estrogenic tropical herb, unveiled the severity of Type 1 LQTS caused by KCNQ1-T587M. J. Arrhythm. 37 (4), 1114–1116. 10.1002/joa3.12576 34386142 PMC8339079

[B81] Kim D. H. Jung H. A. Park S. J. Kim J. M. Lee S. Choi J. S. (2010). The effects of daidzin and its aglycon, daidzein, on the scopolamine-induced memory impairment in male mice. Arch. Pharm. Res. 33, 1685–1690. 10.1007/s12272-010-1019-2 21052945

[B82] Kitisripanya T. Jutathis K. Inyai C. Komaikul J. Udomsin O. Yusakul G. (2016). Anti-miroestrol polyclonal antibodies: a comparison of immunogen preparations used to obtain desired antibody properties. J. Nat. Med. 70, 296–299. 10.1007/s11418-015-0949-x 26563142

[B83] Kitisripanya T. Inyai C. Komaikul J. Krittanai S. Juengwatanatrakul T. Sakamoto S. (2017). A lateral flow colloidal gold-based immunoassay for rapid detection of miroestrol in samples of White Kwao Krua, a phytoestrogen-rich plant. J. Nat. Med. 71, 659–664. 10.1007/s11418-017-1096-3 28573485

[B84] Kitisripanya T. Jutathis K. Inyai C. Komaikul J. Udomsin O. Tanaka H. (2017a). Development of an enzyme-linked immunosorbent assay for the detection of isomiroestrol, an identical marker, in White Kwao Krua using a monoclonal antibody. J. Pharm. Biomed. Anal. 137, 229–234. 10.1016/j.jpba.2017.01.040 28152387

[B85] Kitisripanya T. Udomsin O. Komaikul J. Inyai C. Limsuwanchote S. Yusakul G. (2018). A pilot pharmacokinetic study of miroestrol and deoxymiroestrol on rabbit sera using polyclonal antibody-based icELISA analysis. Phytother. Res. 32, 365–369. 10.1002/ptr.5991 29168310

[B86] Kittivanichkul D. Charoenphandhu N. Khemawoot P. Malaivijitnond S. (2016). *Pueraria mirifica* alleviates cortical bone loss in naturally menopausal monkeys. J. Endocrinol. 231, 121–133. 10.1530/JOE-16-0277 27601445

[B87] Kongkaew C. Scholfield N. C. Dhippayom T. Dilokthornsakul P. Saokaew S. Chaiyakunapruk N. (2018). Efficacy and safety of *Pueraria candollei var. mirifica* (Airy Shaw and Suvat.) Niyomdham for menopausal women: a systematic review of clinical trials and the way forward. J. Ethnopharmacol. 216, 162–174. 10.1016/j.jep.2018.01.028 29409850

[B88] Korsangruang S. Soonthornchareonnon N. Chintapakorn Y. Saralamp P. Prathanturarug S. (2010). Effects of abiotic and biotic elicitors on growth and isoflavonoid accumulation in *Pueraria candollei var. candollei* and *P. candollei var. mirifica* cell suspension cultures. Plant Cell Tissue Organ Cult. 103, 333–342. 10.1007/s11240-010-9785-6

[B89] Krittanai S. Kitisripanya T. Udomsin O. Tanaka H. Sakamoto S. Juengwatanatrakul T. (2018). Development of a colloidal gold nanoparticle-based immunochromatographic strip for the one-step detection of miroestrol and puerarin. Biomed. Chromatogr. 32, e4330. 10.1002/bmc.4330 29972702

[B90] Kulczyński B. Gramza-Michałowska A. Suliburska J. Sidor A. (2021). Puerarin—An isoflavone with beneficial effects on bone health. Front. Biosci. 26, 1653–1667. 10.52586/5058 34994179

[B91] Laddha A. P. Kulkarni Y. A. (2021). Daidzein ameliorates diabetic retinopathy in experimental animals. Life Sci. 265, 118779. 10.1016/j.lfs.2020.118779 33217441

[B92] Lamlertkittikul S. Chandeying V. (2004). Efficacy and safety of *Pueraria mirifica* (Kwao Kruea Khao) for the treatment of vasomotor symptoms in perimenopausal women: phase II study. J. Med. Assoc. Thai 87, 33–40. 14971532

[B93] Lapcharoen P. Apiwathnasorn C. Komalamisra N. Dekumyoy P. Palakul K. Rongsriyam Y. (2005). Three indigenous Thai medicinal plants for control of *Aedes aegypti* and *Culex quinquefasciatus* . Southeast Asian J. Trop. Med. Public Health 36 (Suppl. 4), 167–175. 16438204

[B94] Lee Y. S. Park J. S. Cho S. D. Son J. K. Cherdshewasart W. Kang K. S. (2002). Requirement of metabolic activation for estrogenic activity of *Pueraria mirifica* . J. Vet. Sci. 3, 273–277. 12819377

[B95] Lee J. H. Kim J. Y. Cho S. H. Jeong J. H. Cho S. Park H. J. (2017). Determination of miroestrol and isomiroestrol from *Pueraria mirifica* (White Kwao Krua) in dietary supplements by LC-MS/MS and LC-Q-Orbitrap/MS. J. Chromatogr. Sci. 55, 214–221. 10.1093/chromsci/bmw171 28115391

[B96] Li L. Xue Z. Chen L. Chen X. Wang H. Wang X. (2017). Puerarin suppression of Aβ1–42-induced primary cortical neuron death is largely dependent on ERβ. Brain Res. 1657, 87–94. 10.1016/j.brainres.2016.11.023 27923632

[B97] Li M. Liu B. Xian M. Wang S. Liu P. (2025). Bioinformatics combined with network pharmacology and experimental validation to identify key biomarkers of hepatocellular carcinoma and corresponding compounds in Radix Astragali and *Pueraria mirifica* . Naunyn Schmiedeb. Arch. Pharmacol. 398, 5351–5371. 10.1007/s00210-024-03597-4 39549064

[B211] Li X. Ji W. Wu S. Qian C. Zhou J. Zhang Z. (2024). The isolation, characterization and biological activities of the non-glucan polysaccharides from the high-starch-content plant Pueraria mirifica. Int. J. Biol. Macromol. 261, 129709. 10.1016/j.ijbiomac.2024.129709 38286380

[B98] Li Y. Huang F. Guo Q. Qian X. Liu C. Wang Z. (2025a). Exploring the anti-aging potential of phytoestrogens: focus on molecular mechanisms and menopausal symptom modulation. Front. Nutr. 12, 1651367. 10.3389/fnut.2025.1651367 41080187 PMC12507604

[B99] Liangdeng W. Fengrui Y. Weifeng Z. Ming Z. Xufeng X. Yuekeng Y. (2024). Transcriptomics integrated with targeted metabolomics reveals endogenous hormone changes in tuberous root expansion of Pueraria. BMC Genomics 25, 1112. 10.1186/s12864-024-11010-w 39563238 PMC11577955

[B212] Liga S. Paul C. (2024). Puerarin-a promising flavonoid: biosynthesis, extraction methods, analytical techniques, and biological effects. Int. J. Mol. Sci. 25 (10), 5222. 10.3390/ijms25105222 38791264 PMC11121215

[B100] Lin T. C. Wang K. H. Kao A. P. Chuang K. H. Kuo T. C. (2017). *Pueraria mirifica* inhibits 17β-estradiol-induced cell proliferation of human endometrial mesenchymal stem cells. Taiwan J. Obstet. Gynecol. 56, 765–769. 10.1016/j.tjog.2017.10.011 29241917

[B101] Liu X. Ye H. Dai L. Liu Q. (2015). Studies on the chemical constituents of *Pueraria lobata* from Thailand [in Chinese]. Northwest J. Pharm. 30, 664–666.

[B102] Liu X. Zhao W. Wang W. Lin S. Yang L. (2017). Puerarin suppresses LPS-induced breast cancer cell migration, invasion and adhesion by blockage NF-κB and Erk pathway. Biomed. Pharmacother. 92, 429–436. 10.1016/j.biopha.2017.05.102 28558356

[B103] Loutchanwoot P. Vortherms T. (2018). Effects of puerarin on estrogen-regulated gene expression in gonadotropin-releasing hormone pulse generator of ovariectomized rats. Steroids 135, 54–62. 10.1016/j.steroids.2018.05.003 29733861

[B104] Loutchanwoot P. Vortherms T. Jarry H. (2016). Evaluation of *in vivo* estrogenic potency of natural estrogen-active chemical, puerarin, on pituitary function in gonadectomized female rats. Life Sci. 165, 75–82. 10.1016/j.lfs.2016.09.002 27615593

[B105] Luo J. Li L. Shi W. Xu K. Shen Y. Dai B. (2025). Oxidative stress and inflammation: roles in osteoporosis. Front. Immunol. 16, 1611932. 10.3389/fimmu.2025.1611932 40873591 PMC12379731

[B106] Major D. Johnson D. Tanner J. Anderson I. (1975). Effects of daylength and temperature on soybean development. Crop Sci. 15, 174–179. 10.2135/cropsci1975.0011183x001500020009x

[B107] Makkliang F. Juengsanguanpornsuk W. Phaisan S. Sakdamas A. Putalun W. Sakamoto S. (2021). Transformation of *Pueraria candollei var. mirifica* phytoestrogens using immobilized and free β-glucosidase, a technique for enhancing estrogenic activity. RSC Adv. 11, 32067–32076. 10.1039/D1RA05109A 35495490 PMC9042063

[B108] Malaivijitnond S. (2012). Medical applications of phytoestrogens from the Thai herb *Pueraria mirifica* . Front. Med. 6, 8–21. 10.1007/s11684-012-0184-8 22460444

[B109] Malaivijitnond S. Kiatthaipipat P. Cherdshewasart W. Watanabe G. Taya K. (2004). Different effects of *Pueraria mirifica*, a herb containing phytoestrogens, on LH and FSH secretion in gonadectomized female and male rats. J. Pharmacol. Sci. 96, 428–435. 10.1254/jphs.fpj04029x 15599108

[B110] Malaivijitnond S. Chansri K. Kijkuokul P. Urasopon N. Cherdshewasart W. (2006). Using vaginal cytology to assess the estrogenic activity of phytoestrogen-rich herb. J. Ethnopharmacol. 107, 354–360. 10.1016/j.jep.2006.03.026 16730147

[B111] Malaivijitnond S. Tungmunnithum D. Gittarasanee S. Kawin K. Limjunyawong N. (2010). Puerarin exhibits weak estrogenic activity in female rats. Fitoterapia 81, 569–576. 10.1016/j.fitote.2010.01.019 20117180

[B112] Manonai J. Chittacharoen A. Theppisai U. Theppisai H. (2007). Effect of *Pueraria mirifica* on vaginal health. Menopause 14, 919–924. 10.1097/gme.0b013e3180399486 17415017

[B113] Manonai J. Chittacharoen A. Udomsubpayakul U. Theppisai H. Theppisai U. (2008). Effects and safety of *Pueraria mirifica* on lipid profiles and biochemical markers of bone turnover rates in healthy postmenopausal women. Menopause 15, 530–535. 10.1097/gme.0b013e31815c5fd8 18202589

[B114] Manonai J. Seif C. Böhler G. Jünemann K. P. (2009). The effect of *Pueraria mirifica* on cytologic and urodynamic findings in ovariectomized rats. Menopause 16, 350–356. 10.1097/gme.0b013e318188b279 19098688

[B115] Manosroi A. Manosroi J. (2005). Determination of bioactive compounds in roots of different ages *Pueraria mirifica*, Airy Shaw Suvatabhandhu and *Butea superba*, Roxb. from various locations in Thailand. Traditional Med. and Nutraceuticals 680, 135–138. 10.17660/actahortic.2005.678.18

[B116] Manosroi A. Chaikul P. Chankhampan C. Ruksiriwanich W. Manosroi W. Manosroi J. (2018). 5α-Reductase inhibition and melanogenesis induction of the selected Thai plant extracts. Chiang Mai J. Sci. 45, 220–236.

[B117] Maruyama T. Kawamura M. Kikura-Hanajiri R. Goda Y. (2014). Botanical origin of dietary supplements labeled as “Kwao Keur,” a folk medicine from Thailand. J. Nat. Med. 68, 220–224. 10.1007/s11418-013-0779-7 23677774

[B118] Masada S. Hosoe J. Arai R. Demizu Y. Hakamatsuka T. Goda Y. (2021). Miroestrol quantification in *Pueraria mirifica* crude drugs and products by single-reference UPLC/PDA/MS using relative molar sensitivities to kwakhurin. Chem. Pharm. Bull. 69, 573–580. 10.1248/cpb.c21-00160 33790074

[B119] Masrudin S. S. Mohamad J. (2015). Preventive effect of *Pueraria mirifica* on testosterone-induced prostatic hyperplasia in Sprague Dawley rats. Andrologia 47, 1153–1159. 10.1111/and.12396 25600492

[B120] Messina M. Barnes S. Setchell K. D. R. (2025). Perspective: isoflavones—intriguing molecules but much remains to be learned about these soybean constituents. Adv. Nutr. 16 (5), 100418. 10.1016/j.advnut.2025.100418 40157603 PMC12245442

[B121] Mikulić M. Atanacković Krstonošić M. Kladar N. Vasiljević S. Katanski S. Mamlić Z. (2024). Phytochemical composition of different red clover genotypes based on plant part and genetic traits. Foods 13 (1), 103. 10.3390/foods13010103 PMC10778848 38201131

[B122] Mohamad J. Masrudin S. S. Alias Z. Muhamad N. A. (2019). The effects of *Pueraria mirifica* extract, diadzein and genistein in testosterone-induced prostate hyperplasia in male Sprague Dawley rats. Mol. Biol. Rep. 46, 1855–1871. 10.1007/s11033-019-04638-5 30710233

[B123] Monthakantirat O. Sukano W. Umehara K. Noguchi H. Chulikhit Y. Matsumoto K. (2014). Effect of miroestrol on ovariectomy-induced cognitive impairment and lipid peroxidation in mouse brain. Phytomedicine 21, 1249–1255. 10.1016/j.phymed.2014.06.012 25172786

[B124] Muangman V. Cherdshewasart W. (2001). Clinical trial of the phytoestrogen-rich herb *Pueraria mirifica* as a crude drug in the treatment of symptoms in menopausal women. Siriraj Med. J. 53, 300–309.

[B125] Namken S. Songvut P. Nuengchamnong N. Kemthong T. Khemawoot P. Malaivijitnond S. (2021). Comparative pharmacokinetics of puerarin alone and in *Pueraria mirifica* extract in female cynomolgus monkeys. Planta Med. 87, 395–403. 10.1055/a-1271-7092 33063303

[B126] Niu H. Li Y. Zhang S. Wang M. Zhang Y. Wang T. (2024). Bone marrow adipose tissue as a critical regulator of postmenopausal osteoporosis: a concise review. Clin. Interv. Aging 19, 1259–1272. 10.2147/CIA.S466446 39011312 PMC11249116

[B127] Okamura S. Sawada Y. Satoh T. Sakamoto H. Saito Y. Sumino H. (2008). *Pueraria mirifica* phytoestrogens improve dyslipidemia in postmenopausal women probably by activating estrogen receptor subtypes. Tohoku J. Exp. Med. 216, 341–351. 10.1620/tjem.216.341 19060449

[B128] Ou S. Liang Q. Leng Y. Luo T. Xu X. Xie H. (2025). Puerarin as a multi-targeted modulator of lipid metabolism: molecular mechanisms, therapeutic potential and prospects for nutritional translation. Front. Nutr. 12, 1598897. 10.3389/fnut.2025.1598897 40756564 PMC12314662

[B129] Phaisan S. Yusakul G. Nuntawong P. Sakamoto S. Putalun W. Morimoto S. (2021). Immunochromatographic assay for the detection of kwakhurin and its application for the identification of *Pueraria candollei var. mirifica* (Airy Shaw and Suvat.) Niyomdham. Phytochem. Anal. 32, 503–511. 10.1002/pca.2998 33020994

[B130] Pitsouni E. Grigoriadis T. Douskos A. Kyriakidou M. Falagas M. E. Athanasiou S. (2018). Efficacy of vaginal therapies alternative to vaginal estrogens on sexual function and orgasm of menopausal women: a systematic review and meta-analysis of randomized controlled trials. Eur. J. Obstet. Gynecol. Reprod. Biol. 229, 45–56. 10.1016/j.ejogrb.2018.08.008 30103082

[B131] Pongkitwitoon B. Boonsnongcheep P. Kitisripanya T. Yusakul G. Sakamoto S. Tanaka H. (2019). Preparation of a highly specific single chain variable fragment antibody targeting miroestrol and its application in quality control of *Pueraria candollei* by enzyme-linked immunosorbent assay. Phytochem. Anal. 30, 600–608. 10.1002/pca.2832 31025473

[B132] Prasansuklab A. Brimson J. M. Tencomnao T. (2020). Potential Thai medicinal plants for neurodegenerative diseases: a review focusing on the anti-glutamate toxicity effect. J. Tradit. Complement. Med. 10, 301–308. 10.1016/j.jtcme.2020.03.003 32670825 PMC7340876

[B133] Rani D. Buranasudja V. Kobtrakul K. De-Eknamkul W. Vimolmangkang S. (2021). Elicitation of *Pueraria candollei* var. *mirifica* suspension cells promises antioxidant potential, implying antiaging activity. Plant Cell Tissue Organ Cult. 145 (1), 29–41. 10.1007/s11240-020-01990-4

[B134] Rani D. Kobtrakul K. De-Eknamkul W. Vimolmangkang S. (2022). Magnetized water: a way to enhance isoflavonoids in cultured *Pueraria candollei var. mirifica* cells. Ind. Crops Prod. 180, 114779. 10.1016/j.indcrop.2022.114779

[B135] Rani D. Kobtrakul K. Vimolmangkang S. (2022a). *Pueraria candollei var. mirifica*: a precious source of pharmaceuticals and cosmeceuticals. Thai J. Pharm. Sci. 46, 1–10. 10.56808/3027-7922.2539

[B136] Ratanachamnong P. Phivthong-Ngam L. Namchaiw P. (2020). Daily White Kwao Krua dietary supplement alleviates LDL oxidative susceptibility, plasma LDL level and improves vasculature in a hypercholesterolemia rabbit model. J. Tradit. Complement. Med. 10, 496–503. 10.1016/j.jtcme.2020.05.001 32953566 PMC7484953

[B137] Rattanapisit K. Kitisripanya T. Konyanee A. Sae-Foo W. Burapapiruin A. Putalun W. (2021). Plant-made antibody against miroestrol: a new platform for expression of full-length immunoglobulin G against small-molecule targets in immunoassays. Plant Cell Rep. 40, 723–733. 10.1007/s00299-021-02670-z 33582859

[B138] Sae-Foo W. Krittanai S. Juengsanguanpornsuk W. Yusakul G. Sakamoto S. Putalun W. (2021). Fragment antigen-binding (Fab) antibody-based lateral flow immunoassay for rapid and sensitive detection of potent phytoestrogen, deoxymiroestrol. J. Nat. Med. 75, 1043–1049. 10.1007/s11418-021-01539-5 34106388

[B139] Saenphet K. Kantaoop P. Saenphet S. Aritajat S. (2005). Mutagenicity of *Pueraria mirifica* Airy Shaw and Suvatabandhu and antimutagenicity of *Thunbergia laurifolia* Linn. Southeast Asian J. Trop. Med. Public Health 36 (Suppl. 4), 238–241. 16438216

[B140] Saetae T. (2024). Osteoporosis in androgen deficient rats and monkeys and preventive effects of *Pueraria mirifica* on bone loss. Doctoral dissertation Bangkok, Thailand: Chulalongkorn University.

[B141] Saetae T. Kemthong T. Jaroenporn S. Malaivijitnond S. (2025). Bone-sparing effects of *Pueraria mirifica* extract on bone loss in androgen-deficient cynomolgus macaques (Macaca fascicularis). Thai J. Pharm. Sci. 49 (3), 6. 10.56808/3027-7922.3149

[B142] Sakamoto S. Eto R. Nuntawong P. Yusakul G. Juengwatanatrakul T. Putalun W. (2020). Kwakhurin-magnetic particles conjugates enable fast enzyme immunoassay for the detection of kwakhurin in *Pueraria candollei* . Phytochem. Anal. 31, 930–936. 10.1002/pca.2964 32542923

[B143] Sakamoto S. Minami K. Nuntawong P. Yusakul G. Putalun W. Tanaka H. (2022). Bioimprinting as a receptor for detection of kwakhurin. Biomolecules 12, 1064. 10.3390/biom12081064 36008958 PMC9405580

[B144] Shen J. G. Yao M. F. Chen X. C. Feng Y. F. Ye Y. H. Tong Z. H. (2009). Effects of puerarin on receptor for advanced glycation end products in nephridial tissue of streptozotocin-induced diabetic rats. Mol. Biol. Rep. 36, 2229–2233. 10.1007/s11033-008-9438-6 19125353

[B145] Shimokawa S. Kumamoto T. Ishikawa T. Takashi M. Higuchi Y. Chaichantipyuth C. (2013). Quantitative analysis of miroestrol and kwakhurin for standardisation of Thai miracle herb Kwao Keur (*Pueraria mirifica*) and establishment of simple isolation procedure for highly estrogenic miroestrol and deoxymiroestrol. Nat. Prod. Res. 27, 371–378. 10.1080/14786419.2012.695370 22708703

[B146] Siangcham T. Saenphet S. Saenphet K. (2010). Estrogen bioassay of *Pueraria mirifica* Airy Shaw and Suvatabandhu. J. Med. Plants Res. 4, 741–744. 10.5897/JMPR10.396

[B147] Singh L. (2025). Daidzein’s potential in halting neurodegeneration: unveiling mechanistic insights. Naunyn Schmiedeb. Arch. Pharmacol. 398, 243–259. 10.1007/s00210-024-03356-5 39158734

[B148] Sookvanichsilp N. Soonthornchareonnon N. Boonleang C. (2008). Estrogenic activity of the dichloromethane extract from *Pueraria mirifica* . Fitoterapia 79, 509–514. 10.1016/j.fitote.2008.05.006 18621111

[B149] Srasri M. Srivilai P. Loutchanwoot P. (2022). Assessment of 28-day oral exposure to *Pueraria candollei var. mirifica* (Fabaceae) roots on pituitary-ovarian axis function and selected metabolic parameters in ovary-intact rats. Toxicol. Rep. 9, 1831–1845. 10.1016/j.toxrep.2022.09.013 36518446 PMC9742950

[B151] Sritonchai C. Manonai J. Sophonsritsuk A. Cherdshewasart W. (2020). Comparison of the effects of *Pueraria mirifica* gel and of placebo gel on the vaginal microenvironment of postmenopausal women with genitourinary syndrome of menopause (GSM). Maturitas 140, 49–54. 10.1016/j.maturitas.2020.06.005 32972635

[B152] Su T. (2017). Comparative study of main Pueraria varieties in Thailand and China.

[B153] Sucontphunt A. De-Eknamkul W. Nimmannit U. Dan Dimitrijevich S. Gracy R. W. (2011). Protection of HT22 neuronal cells against glutamate toxicity mediated by the antioxidant activity of *Pueraria candollei var. mirifica* extracts. J. Nat. Med. 65, 1–8. 10.1007/s11418-010-0442-5 20658198

[B154] Sun C. Du M. Sha S. Wang S. Li L. Hou J. (2025). Puerarin improves MASLD by remodeling intestinal microenvironment to promote mitochondrial fusion and autophagy. J. Pharmacol. Sci. 158, 27–41. 10.1016/j.jphs.2025.03.001 40121054

[B155] Suntichaikamolkul N. Tantisuwanichkul K. Prombutara P. Kobtrakul K. Zumsteg J. Wannachart S. (2019). Transcriptome analysis of *Pueraria candollei var. mirifica* for gene discovery in the biosyntheses of isoflavones and miroestrol. BMC Plant Biol. 19, 581. 10.1186/s12870-019-2205-0 31878891 PMC6933718

[B156] Suntichaikamolkul N. Akashi T. Mahalapbutr P. Sanachai K. Rungrotmongkol T. Bassard J. E. (2023). Daidzein hydroxylation by CYP81E63 is involved in the biosynthesis of miroestrol in *Pueraria mirifica* . Plant Cell Physiol. 64, 64–79. 10.1093/pcp/pcac140 36218384

[B157] Suthon S. Jaroenporn S. Charoenphandhu N. Suntornsaratoon P. Malaivijitnond S. (2016). Anti-osteoporotic effects of *Pueraria candollei var. mirifica* on bone mineral density and histomorphometry in estrogen-deficient rats. J. Nat. Med. 70, 225–233. 10.1007/s11418-016-0965-5 26815435

[B158] Suwanvesh N. Manonai J. Sophonsritsuk A. Cherdshewasart W. (2017). Comparison of *Pueraria mirifica* gel and conjugated equine estrogen cream effects on vaginal health in postmenopausal women. Menopause 24, 210–215. 10.1097/GME.0000000000000742 27749740

[B159] Suwanvijitr T. Kaewmuangmoon J. Cherdshewasart W. Chanchao C. (2010). Morphometric and genetic variation in *Pueraria mirifica* cultivars across Thailand. Pak. J. Bot. 42, 97–109.

[B160] Tadsaichol N. Udomuksorn W. Kumarnsit E. Vongvatcharanon U. Vongvatcharanon S. (2024). Effect of *Pueraria mirifica* subchronic treatments on ameliorating depression by increasing tryptophan hydroxylase immunoreactive neurons *via* estrogen receptors in ovariectomized mice. Sains Malays. 53, 3735–3746. 10.17576/jsm-2024-5311-16

[B161] Tahara S. Ingham J. L. Dziedzic S. Z. (1987). Structure elucidation of kwakhurin, a new prenylated isoflavone from *Pueraria mirifica* roots. Z. Naturforsch. C 45, 510–518.

[B162] Tantipongpiradet A. Monthakantirat O. Vipatpakpaiboon O. Khampukdee C. Umehara K. Noguchi H. (2019). Effects of puerarin on the ovariectomy-induced depressive-like behavior in ICR mice and its possible mechanism of action. Molecules 24, 4569. 10.3390/molecules24244569 31847138 PMC6943479

[B163] Thanonkeo S. Palee T. Thanonkeo P. Klanrit P. (2024). Influence of culture conditions on growth and daidzein and genistein production in hairy root cultures of *Pueraria candollei var. mirifica* . Horticulturae 10, 788. 10.3390/horticulturae10080788

[B164] Tiyasatkulkovit W. Charoenphandhu N. Wongdee K. Thongbunchoo J. Krishnamra N. Malaivijitnond S. (2012). Upregulation of osteoblastic differentiation marker mRNA expression in osteoblast-like UMR106 cells by puerarin and phytoestrogens from *Pueraria mirifica* . Phytomedicine 19, 1147–1155. 10.1016/j.phymed.2012.07.010 22951392

[B165] Tiyasatkulkovit W. Malaivijitnond S. Charoenphandhu N. Havill L. M. Ford A. L. Vandeberg J. L. (2014). *Pueraria mirifica* extract and puerarin enhance proliferation and expression of alkaline phosphatase and type I collagen in primary baboon osteoblasts. Phytomedicine 21, 1498–1503. 10.1016/j.phymed.2014.06.019 25442257 PMC4679364

[B166] Trisomboon H. Malaivijitnond S. Watanabe G. Taya K. (2004). Estrogenic effects of *Pueraria mirifica* on the menstrual cycle and hormone-related ovarian functions in cyclic female cynomolgus monkeys. J. Pharmacol. Sci. 94, 51–59. 10.1254/jphs.94.51 14745118

[B167] Trisomboon H. Malaivijitnond S. Suzuki J. Hamada Y. Watanabe G. Taya K. (2004a). Long-term treatment effects of *Pueraria mirifica* phytoestrogens on parathyroid hormone and calcium levels in aged menopausal cynomolgus monkeys. J. Reprod. Dev. 50, 639–645. 10.1262/jrd.50.639 15647615

[B168] Trisomboon H. Malaivijitnond S. Watanabe G. Taya K. (2005). Ovulation block by *Pueraria mirifica*: a study of its endocrinological effect in female monkeys. Endocrine 26, 33–39. 10.1385/ENDO:26:1:033 15805583

[B169] Trisomboon H. Malaivijitnond S. Cherdshewasart W. Watanabe G. Taya K. (2006). Effect of *Pueraria mirifica* on the sexual skin coloration of aged menopausal cynomolgus monkeys. J. Reprod. Dev. 52, 537–542. 10.1262/jrd.18019 16799265

[B170] Trisomboon H. Malaivijitnond S. Watanabe G. Cherdshewasart W. Taya K. (2006). The estrogenic effect of *Pueraria mirifica* on gonadotrophin levels in aged monkeys. Endocrine 29, 129–134. 10.1385/ENDO:29:1:129 16622301

[B171] Trisomboon H. Malaivijitnond S. Cherdshewasart W. Watanabe G. Taya K. (2007). Assessment of urinary gonadotropin and steroid hormone profiles of female cynomolgus monkeys after treatment with *Pueraria mirifica* . J. Reprod. Dev. 53, 395–403. 10.1262/jrd.18079 17202751

[B172] Tsuji G. Yusa M. Masada S. Yokoo H. Hosoe J. Hakamatsuka T. (2020). Facile synthesis of kwakhurin, a marker compound of *Pueraria mirifica* and its quantitative NMR analysis for standardization as a reagent. Chem. Pharm. Bull. 68, 797–801. 10.1248/cpb.c20-00346 32434998

[B173] Udomsin O. Juengwatanatrakul T. Yusakul G. Putalun W. (2015). Chromene stability: the most potent estrogenic compounds in White Kwao Krua (*Pueraria candollei var. mirifica*) crude extract. J. Funct. Foods 19, 269–277. 10.1016/j.jff.2015.09.036

[B174] Udomsuk N. Hamada Y. Asaoka K. Cherdshewasart W. Malaivijitnond S. (2007). *Pueraria mirifica*, a phytoestrogen-rich herb, prevents bone loss in orchidectomized rats. Maturitas 56, 322–331. 10.1016/j.maturitas.2006.09.007 17101247

[B175] Udomsuk L. Juengwattanatrakul T. Jarukamjorn K. Putalun W. (2011). Increased miroestrol, deoxymiroestrol and isoflavonoid accumulation in callus and cell suspension cultures of *Pueraria candollei var. mirifica* . Acta Physiol. Plant. 34, 1093–1100. 10.1007/s11738-011-0906-6

[B176] Udomsuk L. Jarukamjorn K. Putalun W. Sakuma T. Kawasaki Y. Nemoto N. (2011a). Modified expression of aryl hydrocarbon receptor-related genes by deoxymiroestrol, a phytoestrogen, in mouse hepatocytes in primary culture. J. Ethnopharmacol. 137, 902–908. 10.1016/j.jep.2011.06.047 21777665

[B177] Udomsuk L. Juengwatanatrakul T. Putalun W. Jarukamjorn K. (2011b). Down regulation of gene related sex hormone synthesis pathway in mouse testes by miroestrol and deoxymiroestrol. Fitoterapia 82, 1185–1189. 10.1016/j.fitote.2011.08.005 21856387

[B178] Udomsuk L. Juengwatanatrakul T. Putalun W. Jarukamjorn K. (2012). Bimodal action of miroestrol and deoxymiroestrol, phytoestrogens from *Pueraria candollei var. mirifica*, on hepatic CYP2B9 and CYP1A2 expressions and antilipid peroxidation in mice. Nutr. Res. 32, 45–51. 10.1016/j.nutres.2011.11.003 22260863

[B179] Udomsuk L. Chatuphonprasert W. Monthakantirat O. Churikhit Y. Jarukamjorn K. (2012a). Impact of *Pueraria candollei var. mirifica* and its potent phytoestrogen miroestrol on expression of bone-specific genes in ovariectomized mice. Fitoterapia 83, 1687–1692. 10.1016/j.fitote.2012.09.024 23041523

[B180] Udomsuk L. Juengwatanatrakul T. Putalun W. Jarukamjorn K. (2012b). Suppression of BSEP and MRP2 in mouse liver by miroestrol and deoxymiroestrol isolated from *Pueraria candollei* . Phytomedicine 19, 1332–1335. 10.1016/j.phymed.2012.06.007 23017271

[B213] Urasopon N. Hamada Y. Asaoka K. Cherdshewasart W. Malaivijitnond S. (2007). Pueraria mirifica, a phytoestrogen-rich herb, prevents bone loss in orchidectomized rats. Maturit. 56, 322–331. 10.1016/j.maturitas.2006.09.007 17101247

[B181] Urasopon N. Hamada Y. Asaoka K. Poungmali U. Malaivijitnond S. (2008). Isoflavone content of rodent diets and its estrogenic effect on vaginal cornification in *Pueraria mirifica*-treated rats. ScienceAsia 34, 371–376. 10.2306/scienceasia1513-1874.2008.34.371

[B182] Urasopon N. Hamada Y. Cherdshewasart W. Malaivijitnond S. (2008a). Preventive effects of *Pueraria mirifica* on bone loss in ovariectomized rats. Maturitas 59, 137–148. 10.1016/j.maturitas.2008.01.001 18313241

[B183] Vigneswaran K. Hamoda H. (2022). Hormone replacement therapy—current recommendations. Best. Pract. Res. Clin. Obstet. Gynaecol. 81, 8–21. 10.1016/j.bpobgyn.2021.12.001 35000809

[B184] Virojchaiwong P. Suvithayasiri V. Itharat A. (2011). Comparison of *Pueraria mirifica* 25 and 50 mg for menopausal symptoms. Arch. Gynecol. Obstet. 284, 411–419. 10.1007/s00404-010-1689-5 20872225

[B185] Viscardi G. Back S. Ahmed A. Yang S. Blanco Mejia S. Zurbau A. (2025). Effect of soy isoflavones on measures of estrogenicity: a systematic review and meta-analysis of randomized controlled trials. Adv. Nutr. 16 (1), 100327. 10.1016/j.advnut.2024.100327 39433088 PMC11784794

[B186] Vui D. T. Hang N. T. Huy N. Q. Tung B. T. (2019). Investigation of the estrogenic activity of *Pueraria candollei* var. *mirifica* extract on rats. Int. J. Basic Clin. Pharmacol. 8, 2172–2178. 10.18203/2319-2003.ijbcp20194255

[B187] Walker M. D. Shane E. (2023). Postmenopausal osteoporosis. N. Engl. J. Med. 389 (21), 1979–1991. 10.1056/NEJMcp2307353 37991856

[B188] Wang Y. Wang W. L. Xie W. L. Li L. Z. Sun J. Sun W. J. (2013). Puerarin stimulates proliferation and differentiation and protects against cell death in human osteoblastic MG-63 cells *via* ER-dependent MEK/ERK and PI3K/Akt activation. Phytomedicine 20, 787–796. 10.1016/j.phymed.2013.03.005 23639192

[B189] Wang Y. Yang C. Xie W. L. Zhao Y. W. Li Z. M. Sun W. J. (2014). Puerarin concurrently stimulates osteoprotegerin and inhibits receptor activator of NF-κB ligand (RANKL) and interleukin-6 production in human osteoblastic MG-63 cells. Phytomedicine 21, 1032–1036. 10.1016/j.phymed.2014.04.012 24854571

[B214] Wang Z. Wang T. Hu T. Jiao H. Jin Y. Sun J. (2024). Comparisons of wild and cultivated American ginseng (Panax quinquefolius L.) genomes provide insights into changes in root growth and metabolism during domestication. Plant Biotechnol. J. 22, 1963–1965. 10.1111/pbi.14316 38446695 PMC11182593

[B190] Warinsiriruk P. Tantitham C. Cherdshewasart W. Shobeiri S. A. Manonai J. (2022). Effects of *Pueraria mirifica* on vaginal artery vascularization in postmenopausal women with genitourinary syndrome of menopause. Maturitas 160, 4–10. 10.1016/j.maturitas.2022.01.005 35550707

[B191] Wattanapitayakul S. K. Chularojmontri L. Srichirat S. (2005). Effects of *Pueraria mirifica* on vascular function of ovariectomized rabbits. J. Med. Assoc. Thai 88 (Suppl. 1), S21–S29. 16862667

[B192] Wiriyaampaiwong P. Thanonkeo S. Pornthap T. (2012). Cloning and characterization of chalcone synthase gene from *Pueraria candollei* var. *mirifica* . J. Med. Plants Res. 6, 5469–5479. 10.5897/JMPR12.036

[B193] Wiriyakarun S. Yodpetch W. Komatsu K. Zhu S. Ruangrungsi N. Sukrong S. (2013). Discrimination of the Thai rejuvenating herbs *Pueraria candollei* (White Kwao Khruea), *Butea superba* (Red Kwao Khruea), and *Mucuna collettii* (Black Kwao Khruea) using PCR-RFLP. J. Nat. Med. 67, 562–570. 10.1007/s11418-012-0716-1 23086155

[B194] Wu Z. Wu J. Fang P. Kan S. (2017). Puerarin increases the chemosensitivity of hepatocellular carcinoma cells. Oncol. Lett. 14 (3), 3006–3010. 10.3892/ol.2017.6524 28928838 PMC5588135

[B195] Xu L. Kaopong R. Nualkaew S. Chullasara A. Phongdara A. (2017). Expression and functional analysis of a transgenic cytochrome P450 monooxygenase in *Pueraria mirifica* . Sains Malays. 46, 1491–1498. 10.17576/jsm-2017-4609-18

[B196] Yagi N. Nakahashi H. Kobayashi T. Miyazawa M. (2013). Characteristic chemical components of the essential oil from White Kwao Krua (*Pueraria mirifica*). J. Oleo Sci. 62, 175–179. 10.5650/jos.62.175 23470445

[B197] Yen J.-H. Yang D.-J. Chen M.-C. Wu Y.-Y. Hsieh Y.-F. Cheng Y.-M. (2014). Daidzein enhances efferocytosis *via* transglutaminase 2 and augmentation of Rac1 activity. Mol. Immunol. 60, 135–142. 10.1016/j.molimm.2014.04.006 24859791

[B198] Yu Z. Li W. (2006). Induction of apoptosis by puerarin in colon cancer HT-29 cells. Cancer Lett. 238, 53–60. 10.1016/j.canlet.2005.06.022 16055262

[B199] Yusakul G. Putalun W. Udomsin O. Juengwatanatrakul T. Chaichantipyuth C. (2011). Comparative analysis of the chemical constituents of two varieties of *Pueraria candollei* . Fitoterapia 82, 203–207. 10.1016/j.fitote.2010.09.009 20858535

[B200] Yusakul G. Udomsin O. Juengwatanatrakul T. Tanaka H. Chaichantipyuth C. Putalun W. (2013a). High performance enzyme-linked immunosorbent assay for determination of miroestrol, a potent phytoestrogen from Pueraria candollei. Anal. Chim. Acta 785, 104–110. 10.1016/j.aca.2013.04.053 23764450

[B201] Yusakul G. Udomsin O. Juengwatanatrakul T. Tanaka H. Chaichantipyuth C. Putalun W. (2013b). Highly selective and sensitive determination of deoxymiroestrol using a polyclonal antibody-based enzyme-linked immunosorbent assay. Talanta 114, 73–78. 10.1016/j.talanta.2013.04.011 23953444

[B202] Yusakul G. Sakamoto S. Juengwatanatrakul T. Putalun W. Tanaka H. Morimoto S. (2016). Preparation and application of a monoclonal antibody against the isoflavone glycoside daidzin using a mannich reaction-derived hapten conjugate. Phytochem. Anal. 27, 81–88. 10.1002/pca.2604 26689919

[B203] Yusakul G. Kitisripanya T. Juengwatanatrakul T. Sakamoto S. Tanaka H. Putalun W. (2018). Enzyme linked immunosorbent assay for total potent estrogenic miroestrol and deoxymiroestrol of *Pueraria candollei*, a Thai herb for menopause remedy. J. Nat. Med. 72, 641–650. 10.1007/s11418-018-1194-x 29492802

[B204] Yusakul G. Togita R. Minami K. Chanpokapaiboon K. Juengwatanatrakul T. Putalun W. (2019). An indirect competitive enzyme-linked immunosorbent assay toward the standardization of Pueraria candollei based on its unique isoflavonoid, kwakhurin. Fitoterapia 133, 23–28. 10.1016/j.fitote.2018.12.010 30572086

[B205] Yusakul G. Juengsanguanpornsuk W. Sritularak B. Phaisan S. Juengwatanatrakul T. Putalun W. (2021). (+)-7-O-Methylisomiroestrol, a new chromene phytoestrogen from the *Pueraria candollei* var. *mirifica* root. Nat. Prod. Res. 35, 4110–4114. 10.1080/14786419.2020.1727473 32077760

[B206] Zafar S. Luo Y. Zhang L. Li C. H. Khan A. Khan M. I. (2023). Daidzein attenuated paclitaxel-induced neuropathic pain *via* the down-regulation of TRPV1/P2Y and up-regulation of Nrf2/HO-1 signaling. Inflammopharmacology 31, 1977–1992. 10.1007/s10787-023-01225-w 37145202

[B207] Zhang W. G. Yin X. C. Liu X. F. Meng K. W. Tang K. Huang F. L. (2017). Puerarin induces hepatocellular carcinoma cell apoptosis modulated by MAPK signaling pathways in a dose-dependent manner. Anticancer Res. 37 (8), 4425–4431. 10.21873/anticanres.11837 28739736

[B208] Zhu H. Xing Y. Akan O. D. Yang T. (2023). Ultrafine comminution-assisted ultrasonic-microwave synergistic extraction of *Pueraria mirifica* (Kudzu flower and root) flavonoids. Heliyon 9, e21137. 10.1016/j.heliyon.2023.e21137 37920497 PMC10618490

[B209] Zhu T. He J. Li J. Liu C. Min X. Hu X. (2024). Integrative analyses of metabolome and transcriptome reveal regulatory network of puerarin biosynthesis in *Pueraria montana var. lobata* . Molecules 29, 5556. 10.3390/molecules29235556 39683717 PMC11643513

